# Supplementary material for: Analysis of NIS Plasma Membrane Interactors Discloses Key Regulation by a SRC/RAC1/PAK1/PIP5K/EZRIN Pathway with Potential Implications for Radioiodine Re-Sensitization Therapy in Thyroid Cancer
Source: Cancers (Basel). 2021 Oct 30;13(21):5460. doi: 10.3390/cancers13215460 (PMC8582450; doi:10.3390/cancers13215460)

Analysis of NIS plasma membrane interactors discloses key regulation by a SRC/RAC1/PAK1/PIP5K/EZRIN pathway with potential implications for radioiodine re-sensitization therapy in thyroid cancer

Márcia Faria[1,4,5], Rita Domingues[1,2], Maria João Bugalho[1,3], Ana Luísa Silva[1,2,3,6], Paulo Matos[4,5,6,*]

[1] Serviço de Endocrinologia, Diabetes e Metabolismo do CHULN-Hospital Santa Maria, 1649-028 Lisboa, Portugal.

[2] ISAMB-Instituto de Saúde Ambiental, Faculdade de Medicina da Universidade de Lisboa, 1649-028 Lisboa, Portugal.

[3] Faculdade de Medicina da Universidade de Lisboa, 1649-028 Lisboa, Portugal.

[4] BioISI-Biosystems and Integrative Sciences Institute, Faculdade de Ciências da Universidade de Lisboa, Lisboa, Portugal.

[5] Departamento de Genética Humana, Instituto Nacional de Saúde Doutor Ricardo Jorge, Lisboa, Portugal.

[6] Co-senior authors

* Corresponding author

Original WB films used to assemble the various Figures

Figure 1B

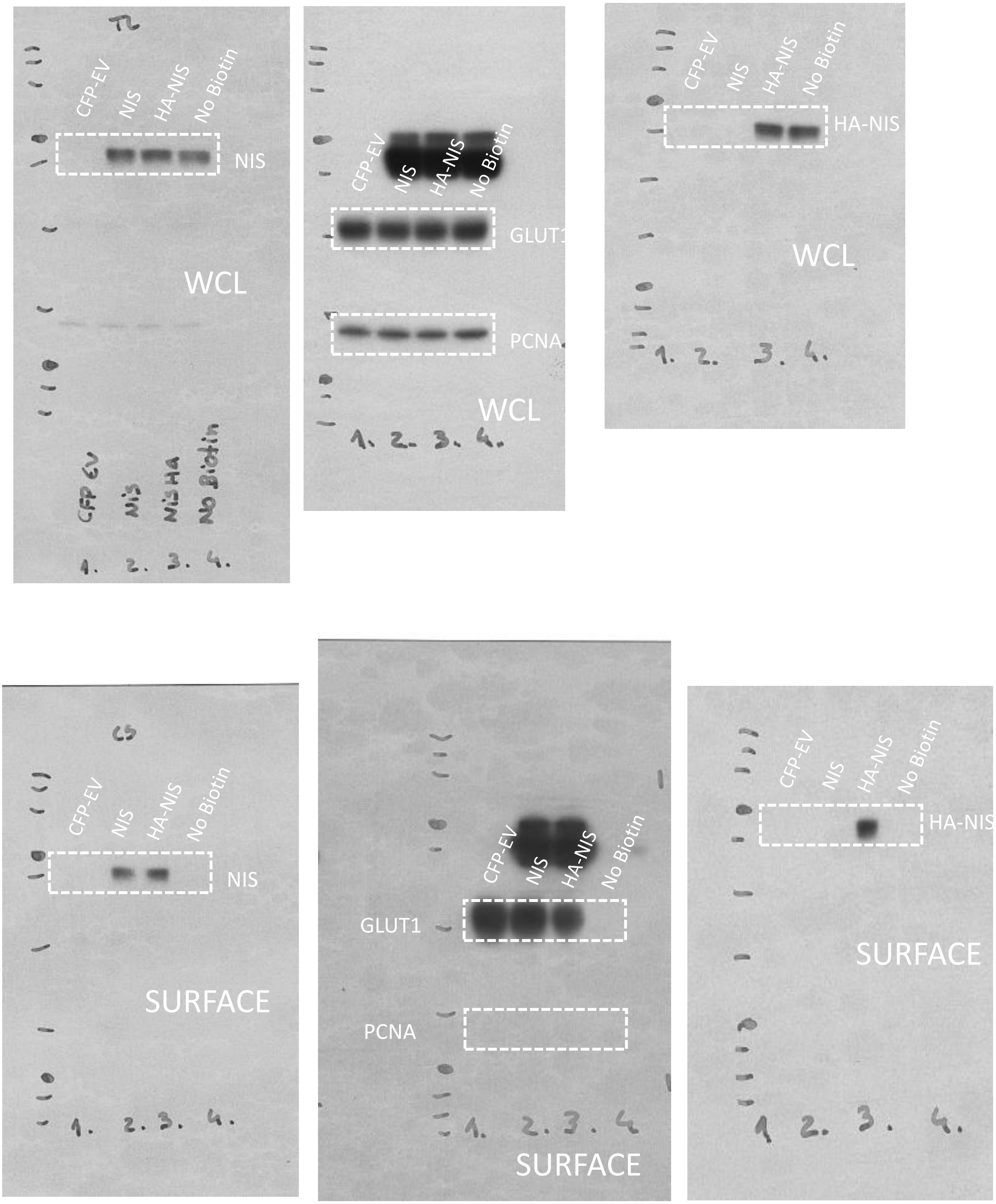
TL
CFP-EV
NIS
HA-NIS
No Biotin
NIS
WCL
CFP EV
NIS
NIS HA
No Biotin
1. 2. 3. 4.
CFP-EV
NIS
HA-NIS
No Biotin
GLUT1
PCNA
WCL
1. 2. 3. 4.
CFP-EV
NIS
HA-NIS
No Biotin
HA-NIS
WCL
1. 2. 3. 4.
CS
CFP-EV
NIS
HA-NIS
No Biotin
NIS
SURFACE
1. 2. 3. 4.
CFP-EV
NIS
HA-NIS
No Biotin
GLUT1
PCNA
1. 2. 3. 4
SURFACE
CFP-EV
NIS
HA-NIS
No Biotin
HA-NIS
SURFACE
1 2. 3. 4.

Figure 2B

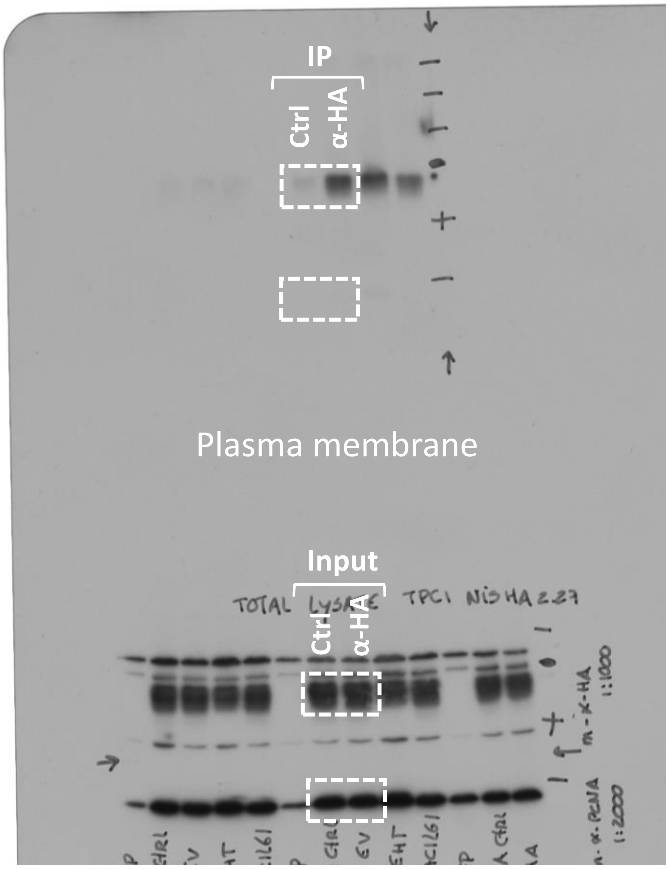
IP
Ctrl
α-HA
Plasma membrane
Input
Ctrl
α-HA

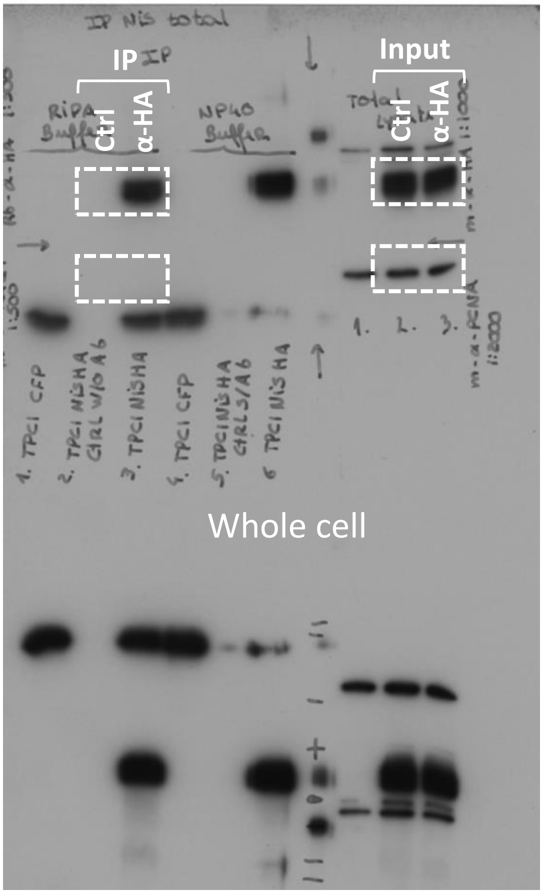
IP
Ctrl
α-HA
Input
Ctrl
α-HA
Whole cell

Figure 3B (left panels)

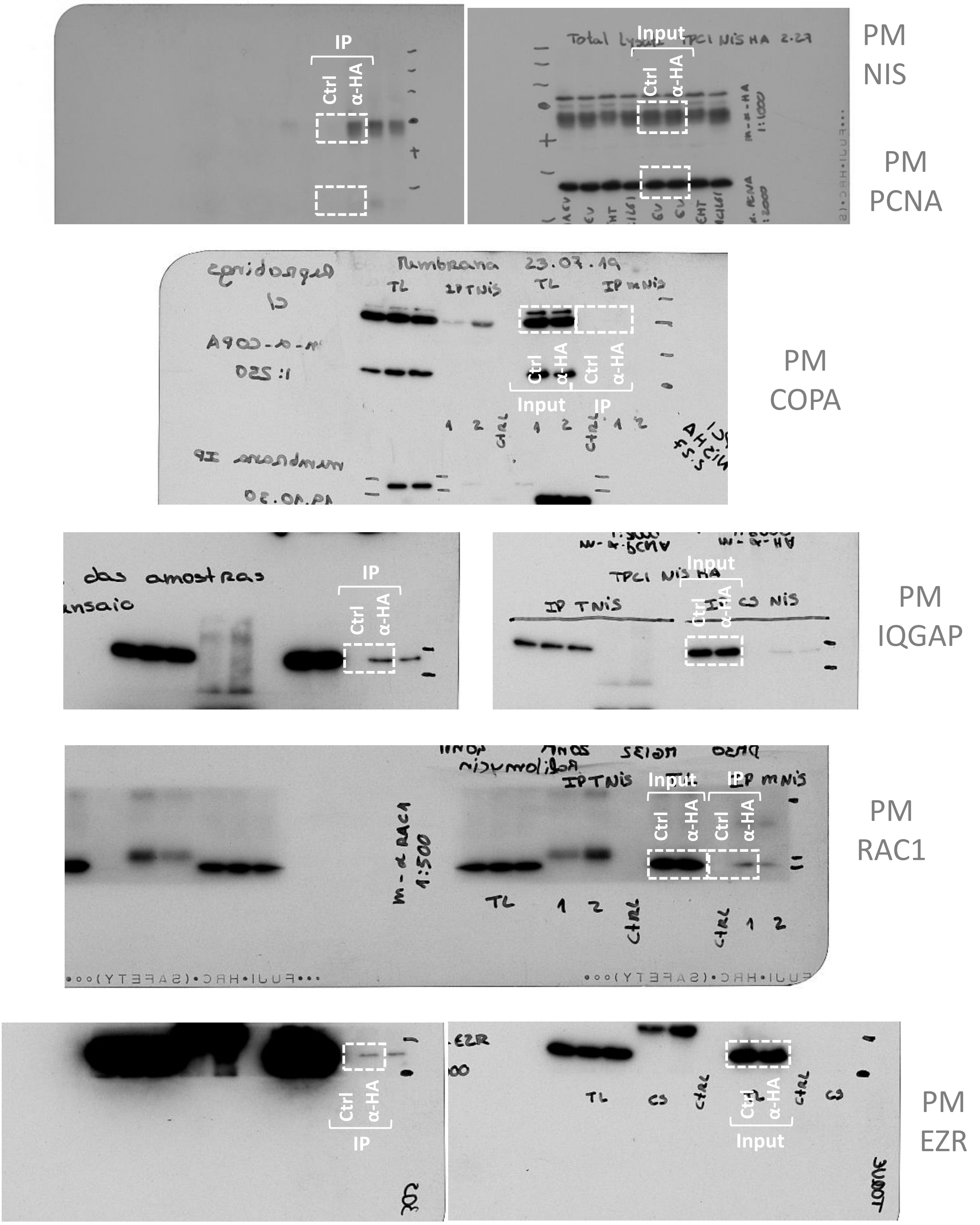

Figure 3B (right panels)

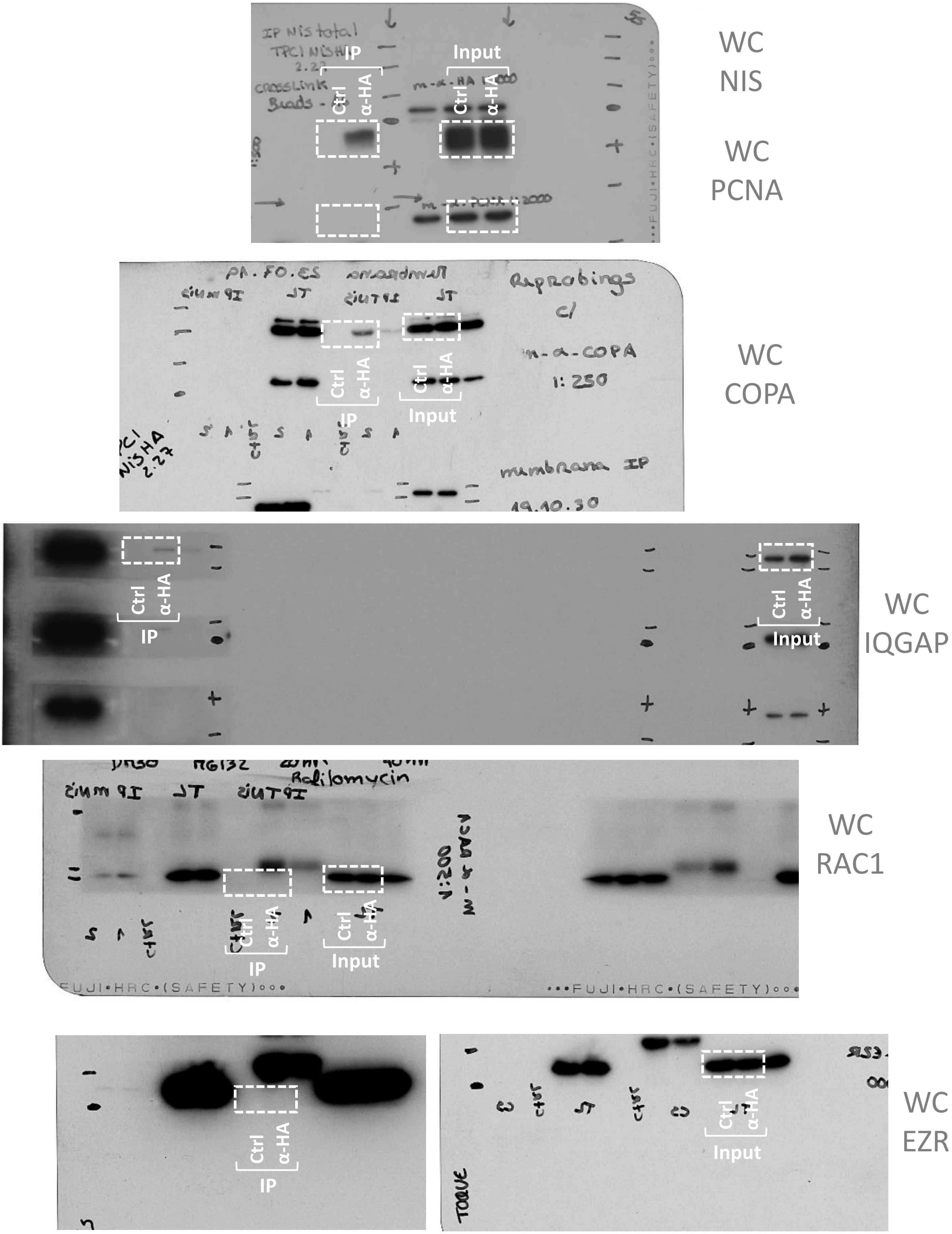

Figure 4A (left panels)

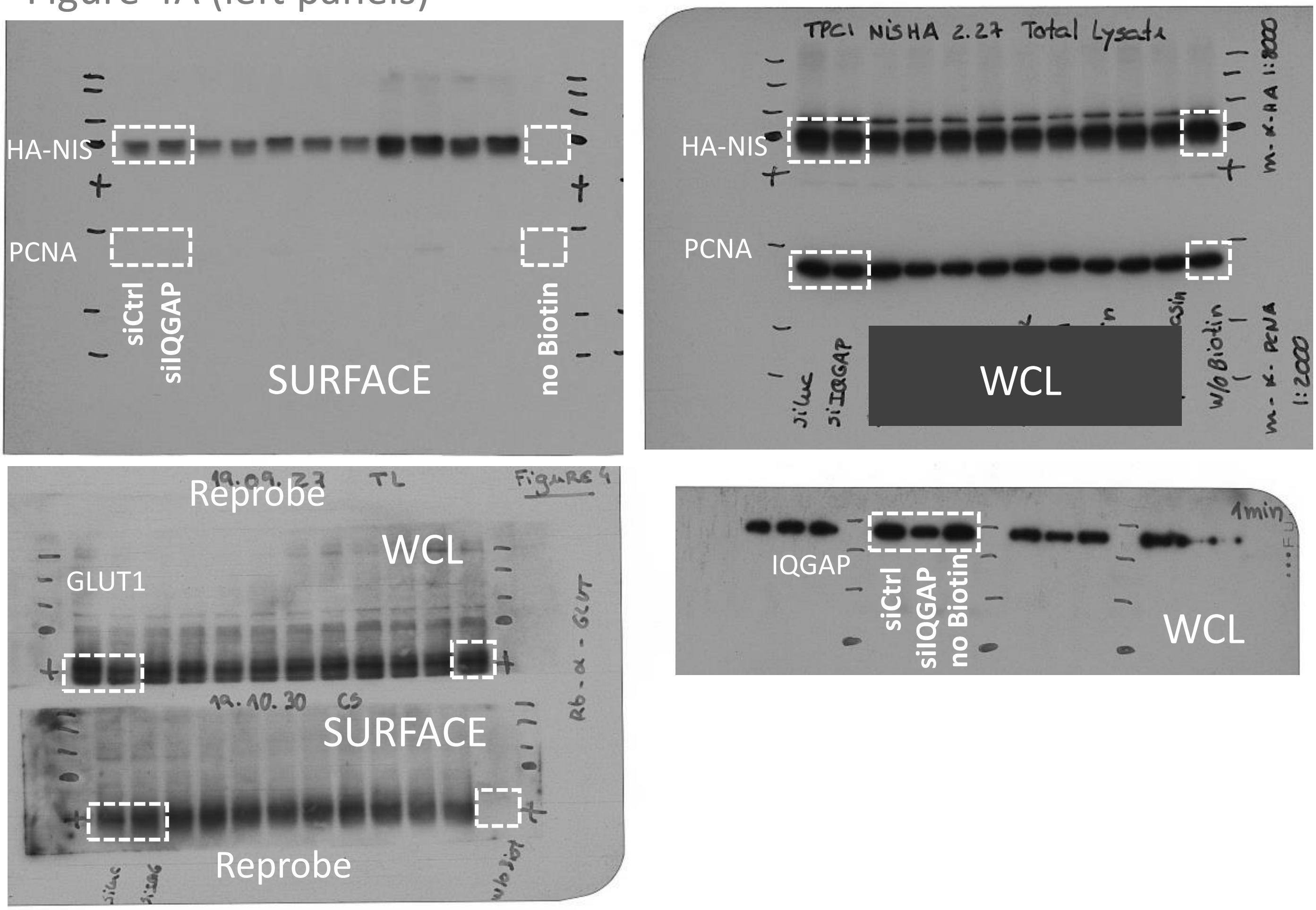

Figure 4A (middle panels)

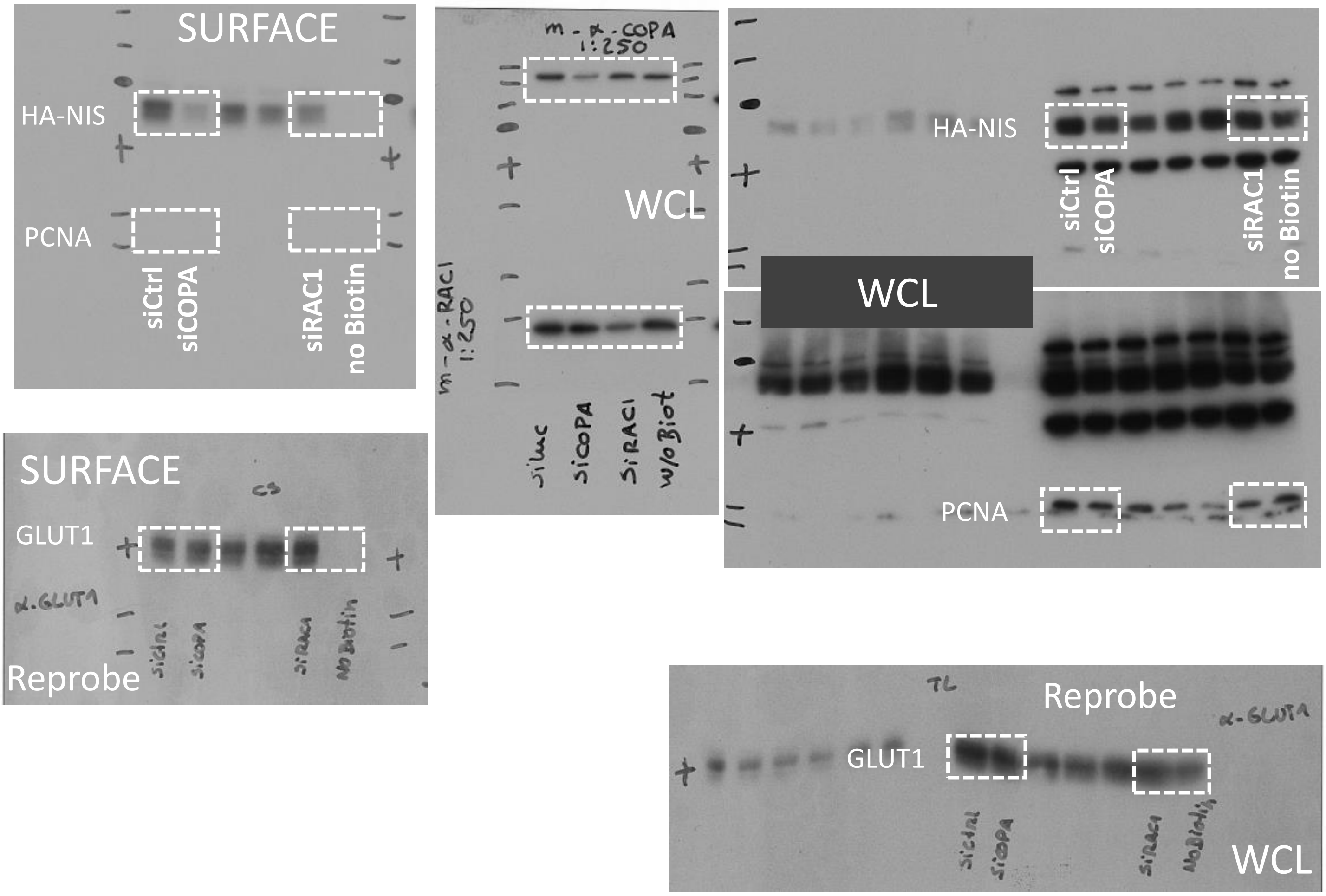

Figure 4A (right panels)

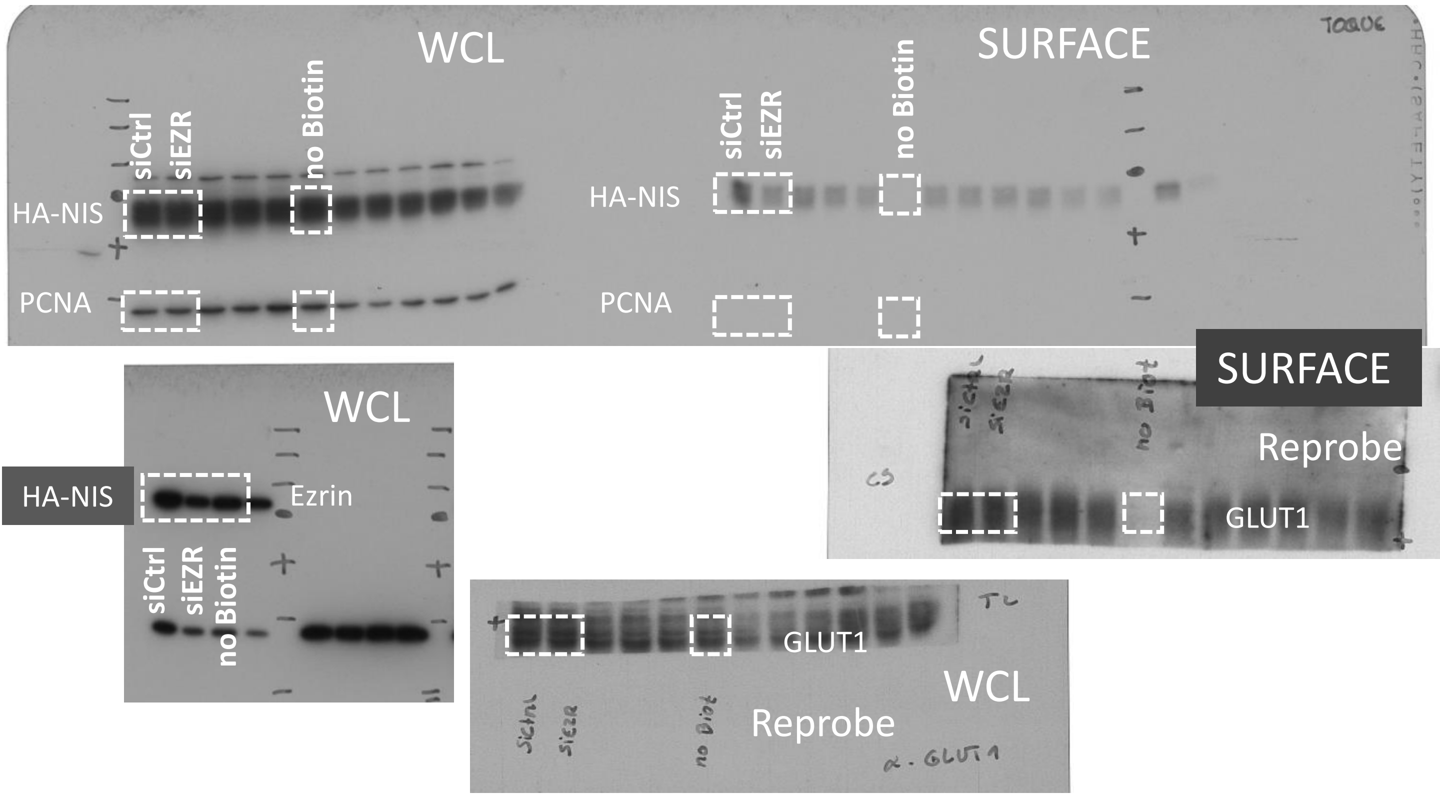

Figure 4C

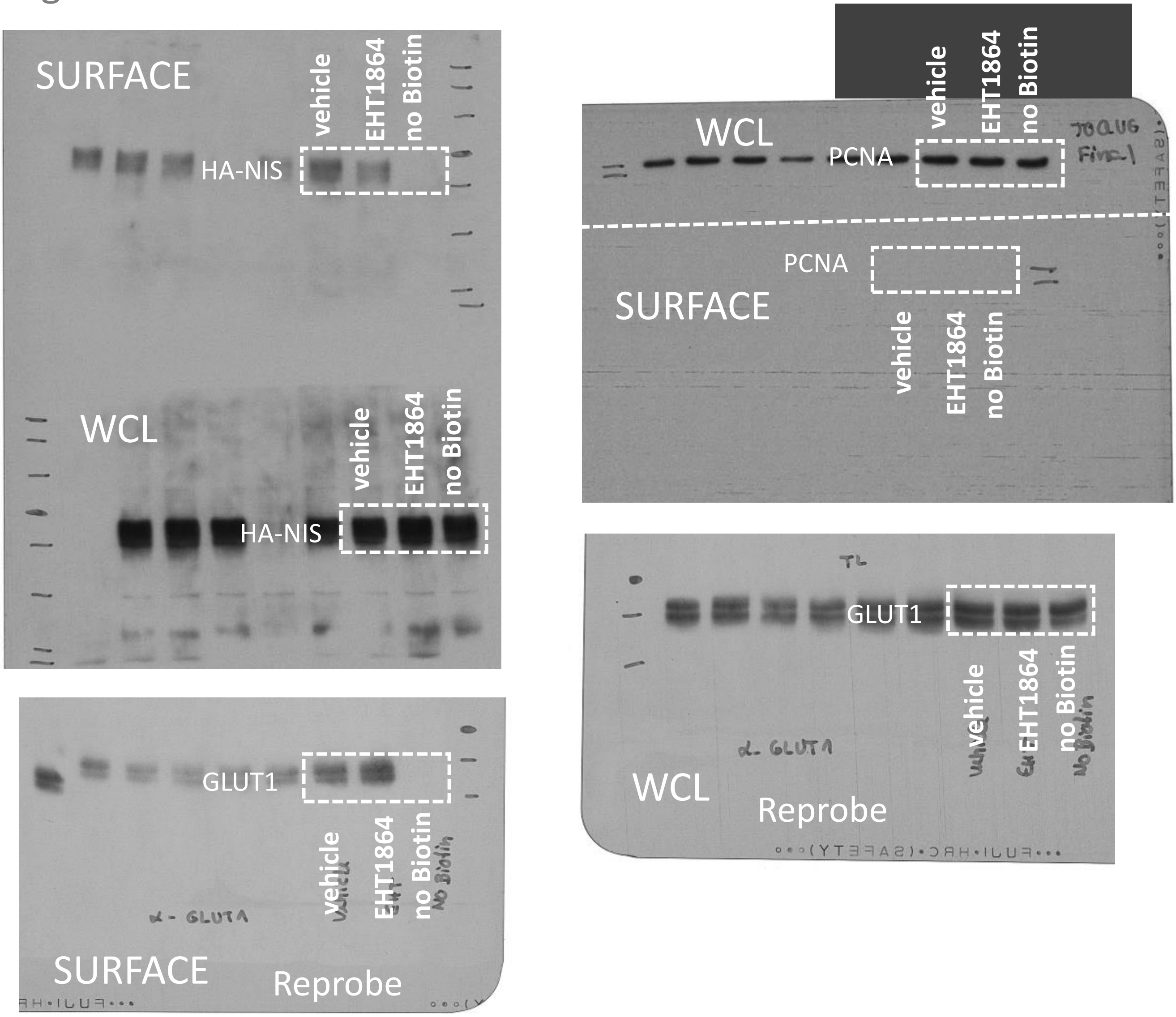

Figure 4E

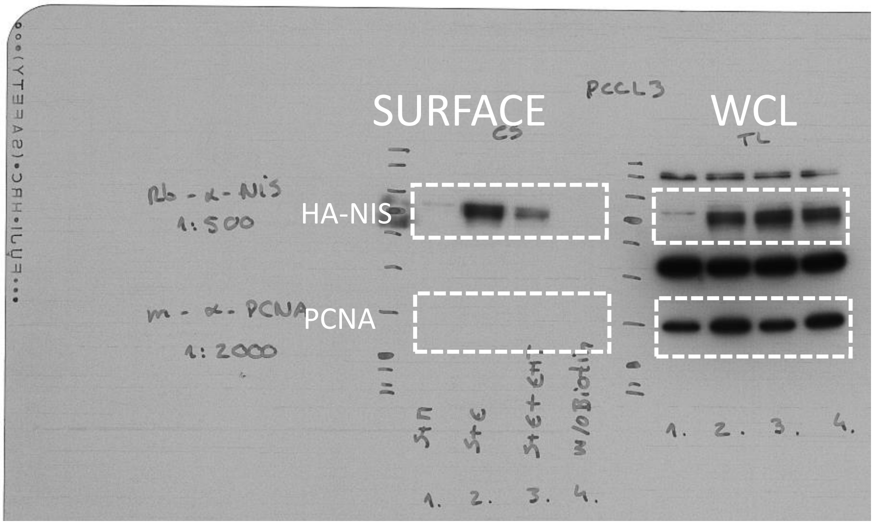

SURFACE
WCL
PCCL3
HA-NIS
PCNA
1:500
1:2000
1. 2. 3. 4.

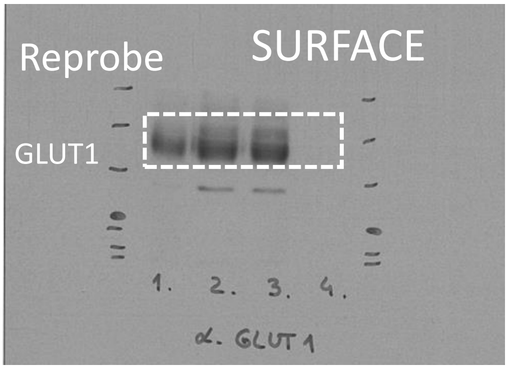

Reprobe
SURFACE
GLUT1
1. 2. 3. 4.

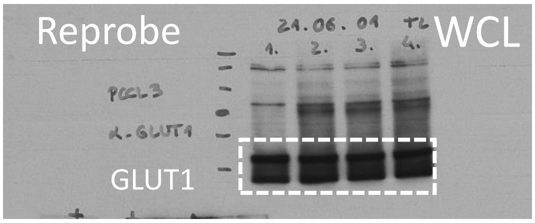

Reprobe
WCL
GLUT1
1. 2. 3. 4.

Supplemental Fig. S3

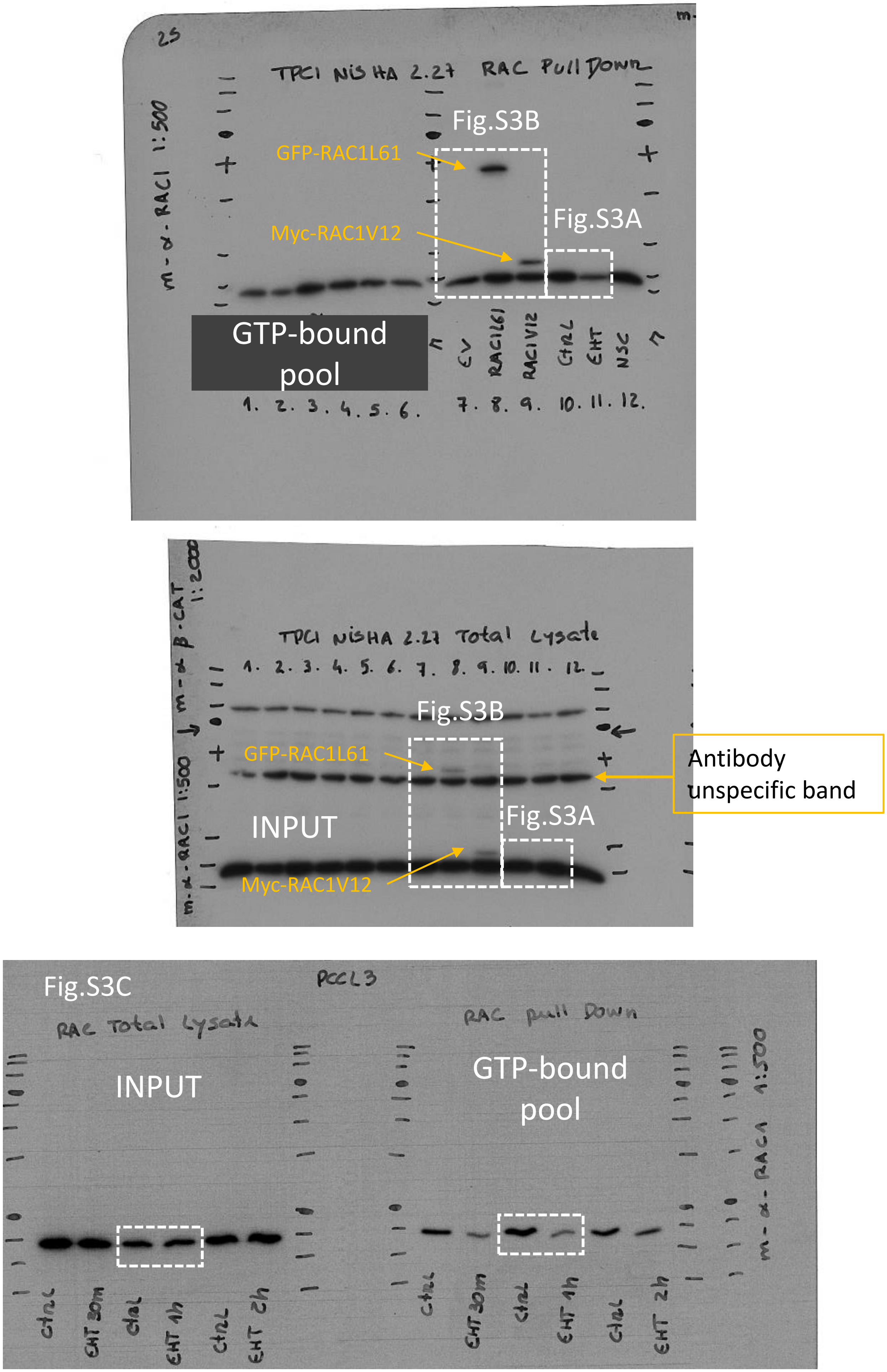

Figure 5A (left panels) (right panels)

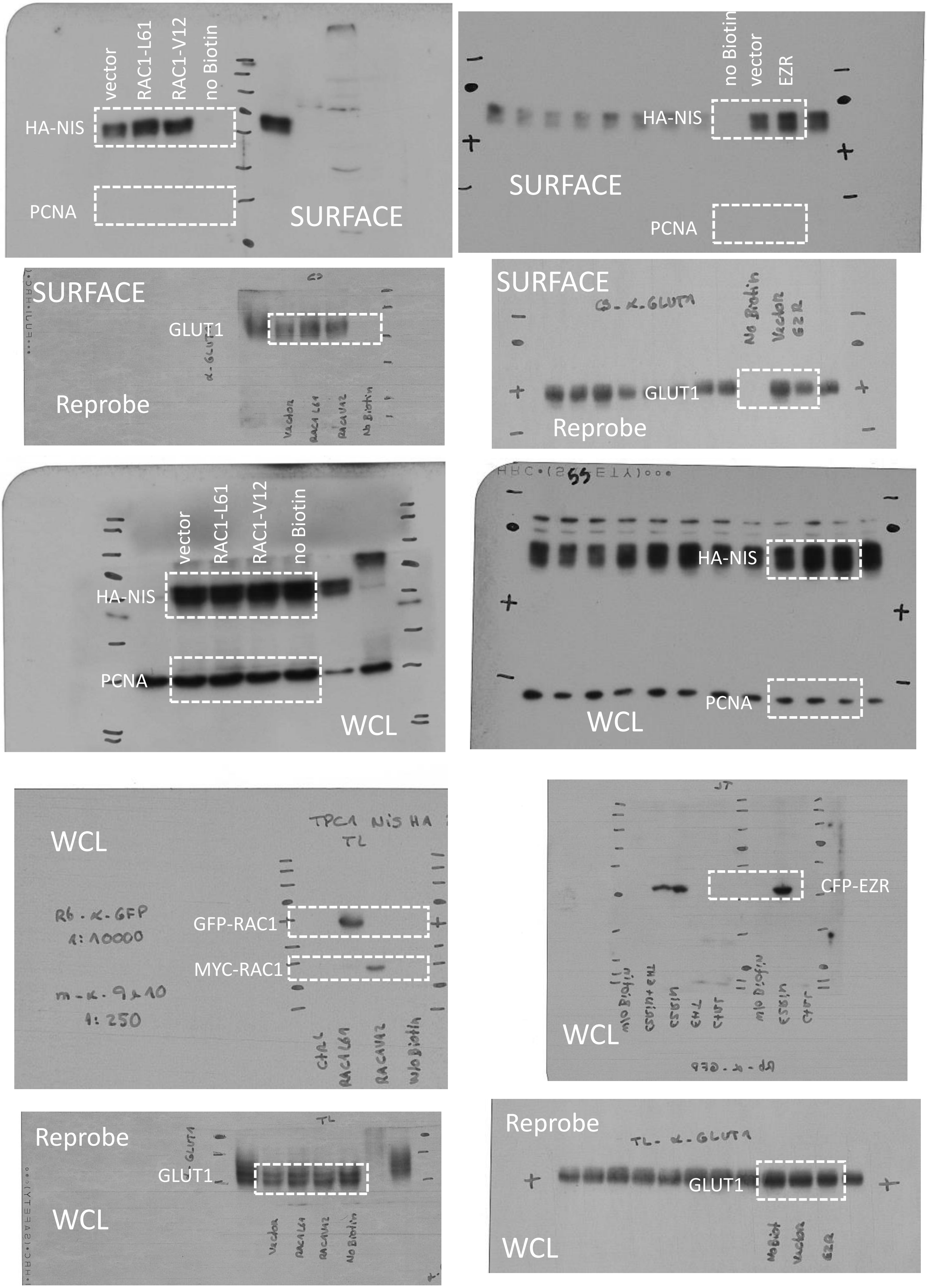

Figure 6A

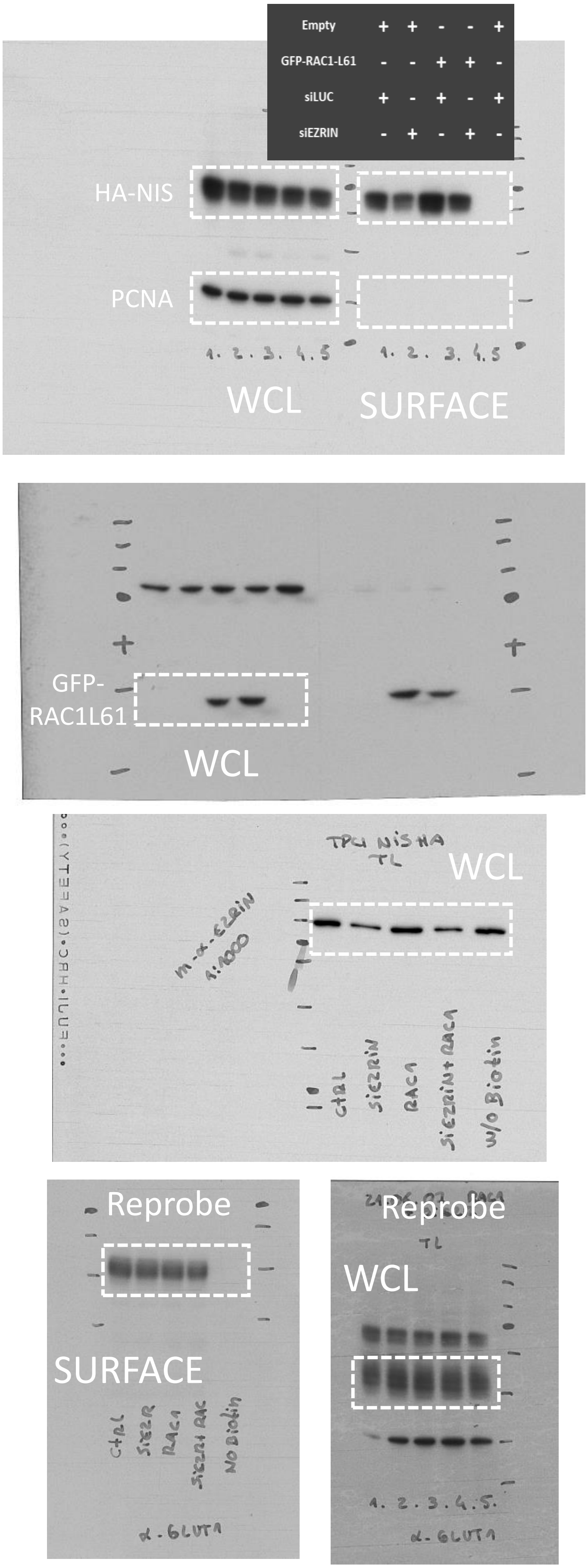

Figure 6B

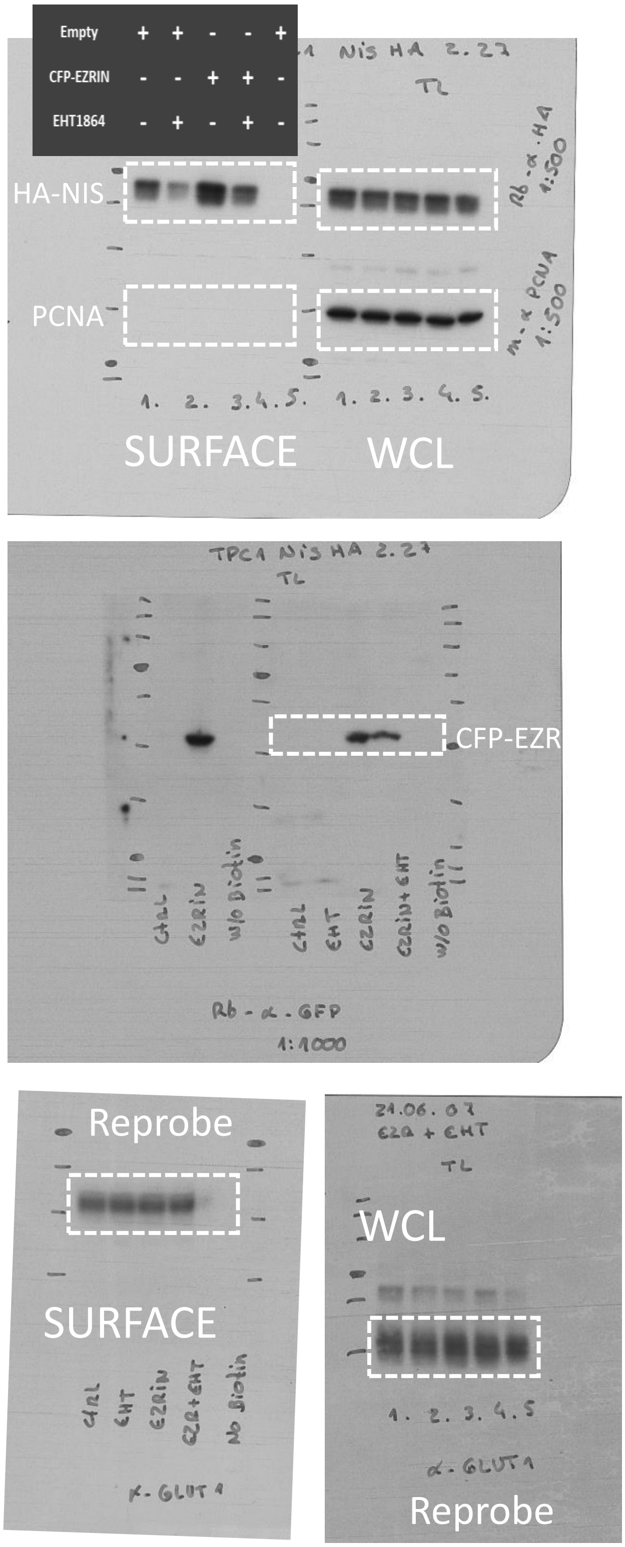

Figure 6C

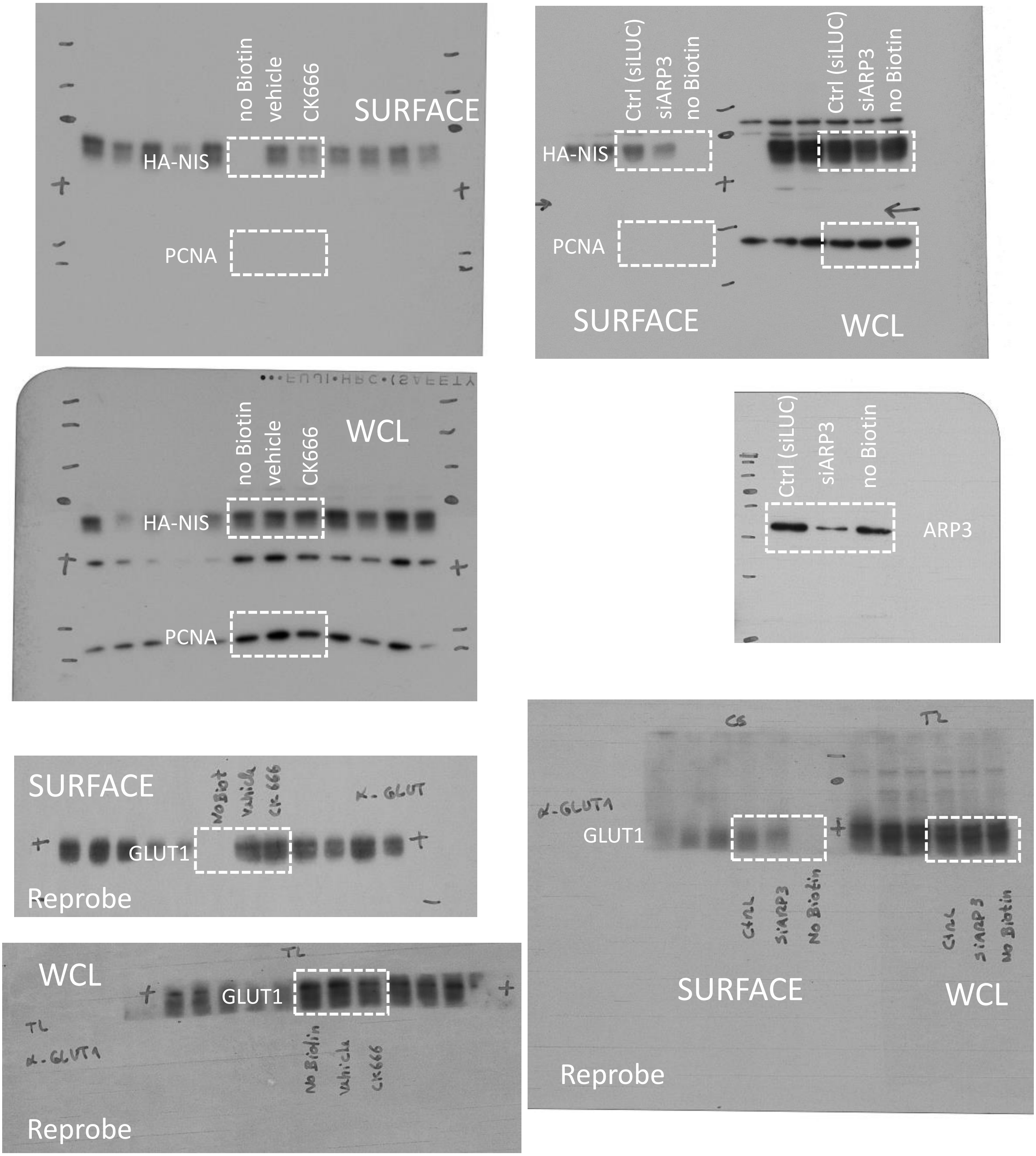

no Biotin
vehicle
CK666
SURFACE
HA-NIS
PCNA
Ctrl (siLUC)
siARP3
no Biotin
Ctrl (siLUC)
siARP3
no Biotin
HA-NIS
PCNA
SURFACE
WCL
no Biotin
vehicle
CK666
WCL
HA-NIS
PCNA
Ctrl (siLUC)
siARP3
no Biotin
ARP3
SURFACE
GLUT1
Reprobe
GLUT1
SURFACE
WCL
Reprobe
WCL
GLUT1
Reprobe

Figure 6D

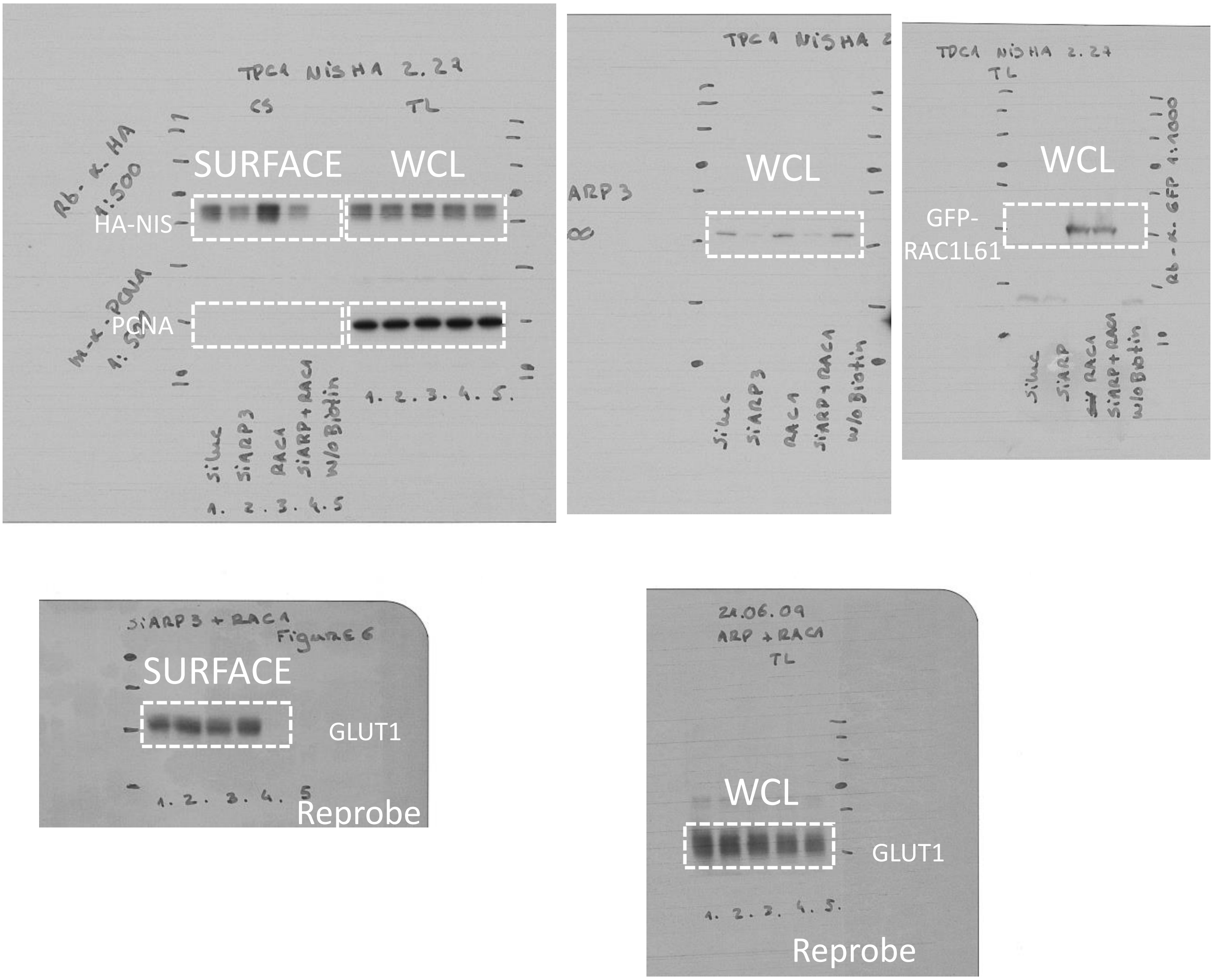

SURFACE
WCL
HA-NIS
PCNA
WCL
WCL
GFP-
RAC1L61
SURFACE
GLUT1
Reprobe
WCL
GLUT1
Reprobe

Supplemental Figure 4A (left panels)

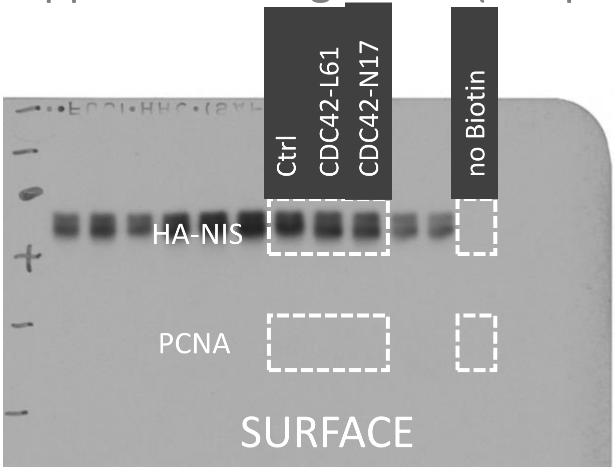

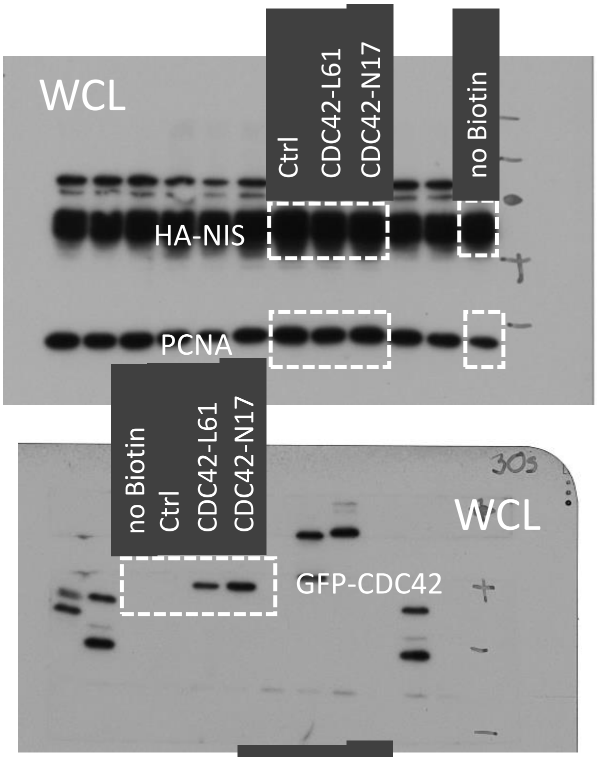

(right panels)

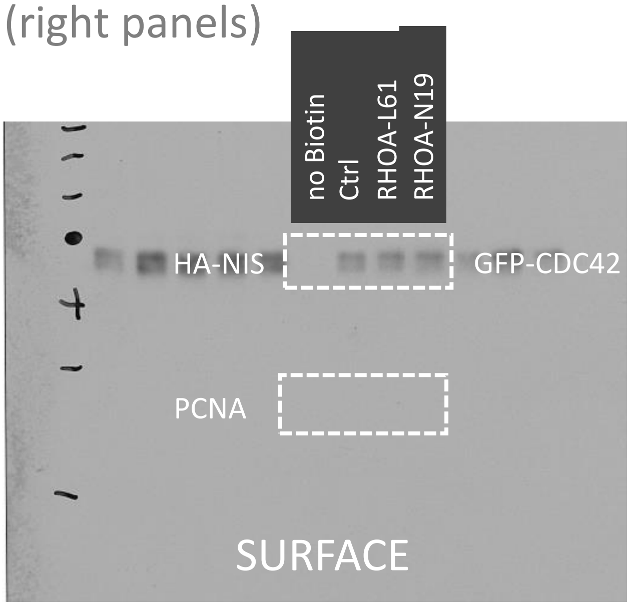

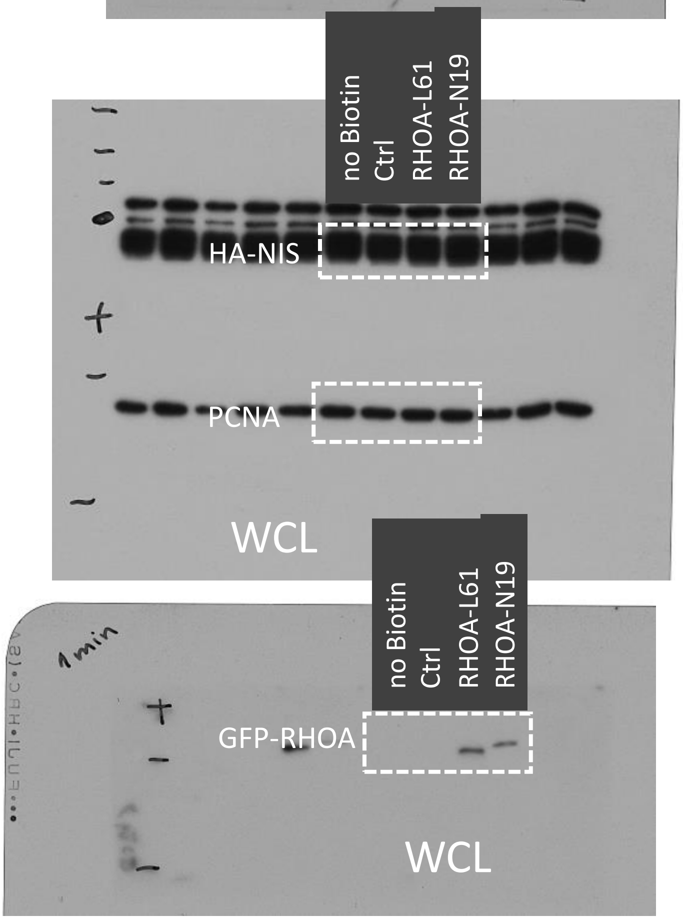

Supplemental Figure 4B

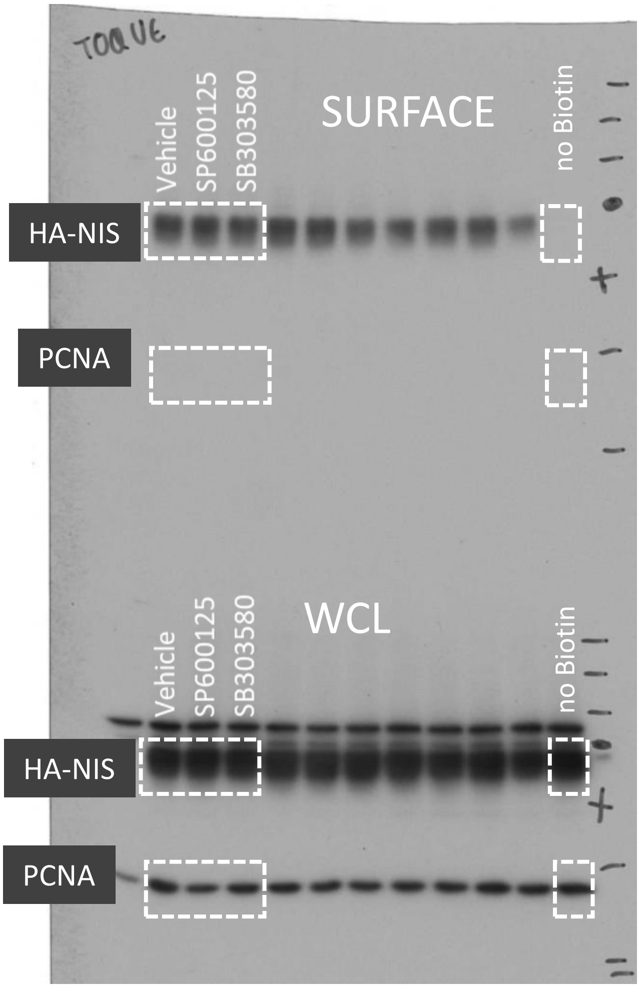

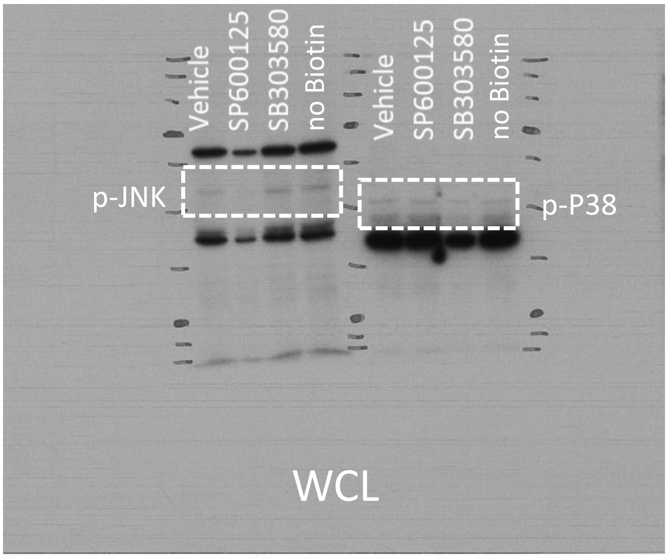

Figure 7A

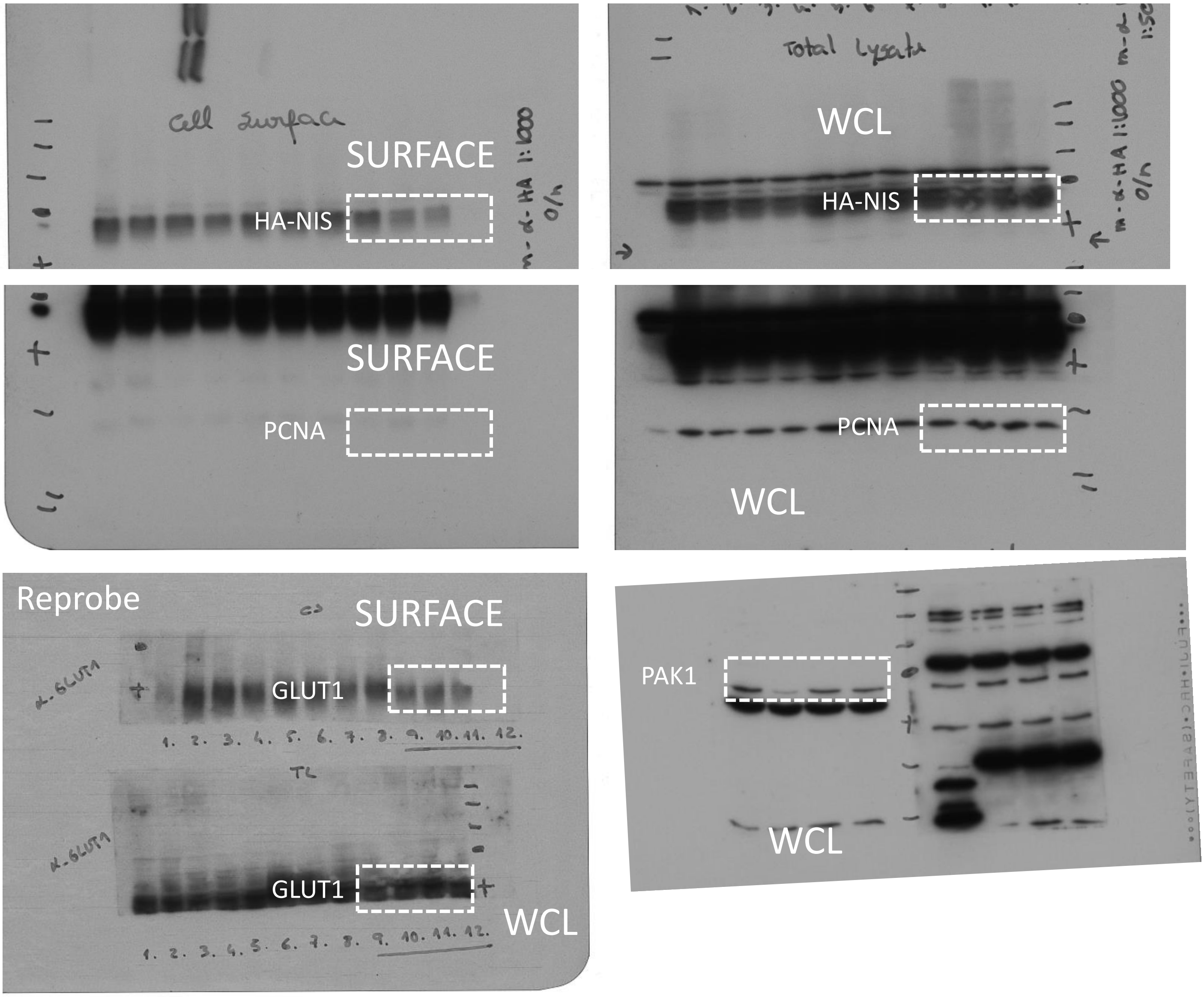
SURFACE
HA-NIS
WCL
HA-NIS
SURFACE
PCNA
PCNA
WCL
Reprobe
SURFACE
GLUT1
PAK1
WCL
GLUT1
WCL

Figure 7B

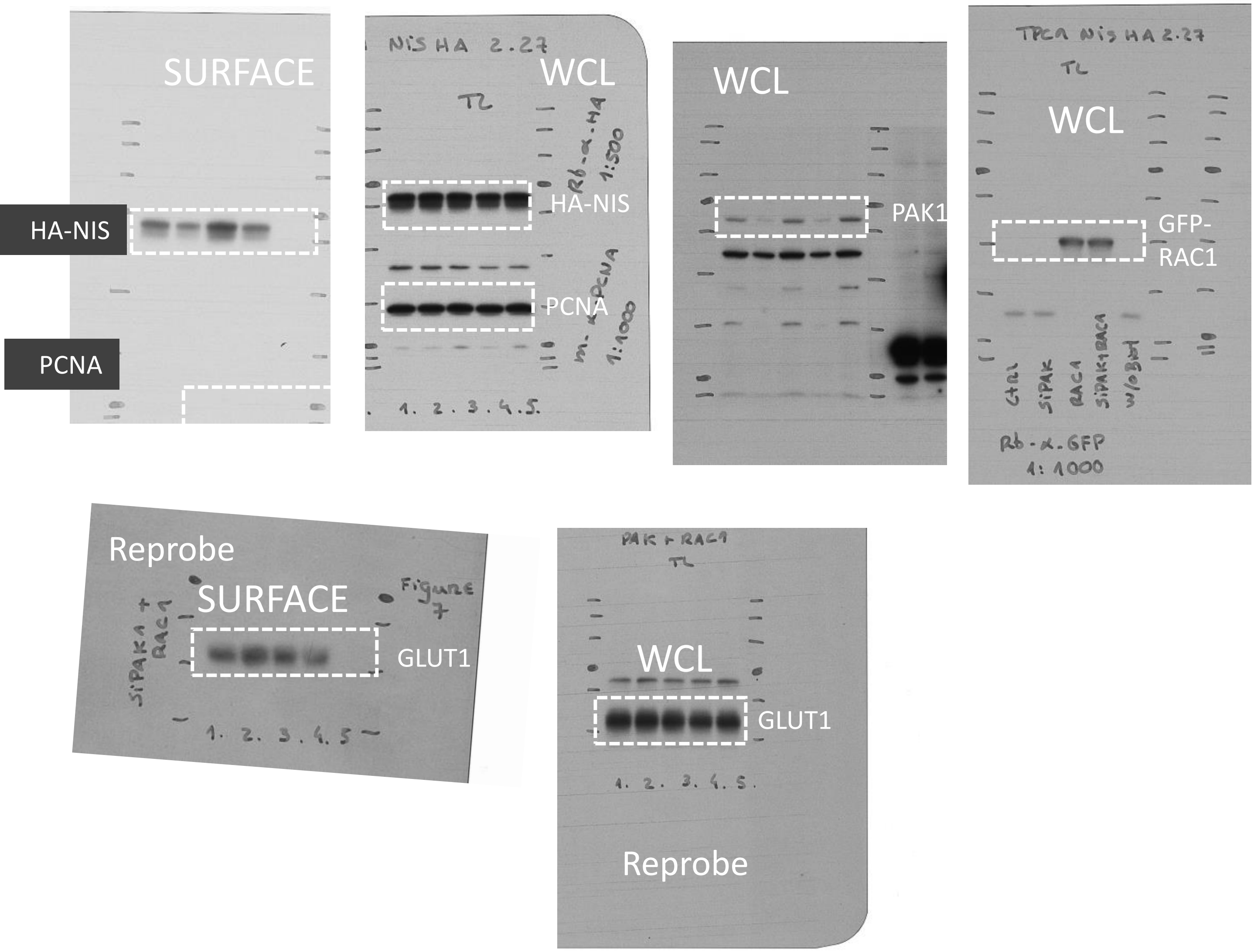
SURFACE
WCL
WCL
WCL
HA-NIS
PCNA
HA-NIS
PCNA
PAK1
GFP-RAC1
Reprobe
SURFACE
GLUT1
WCL
GLUT1
Reprobe

Figure 8A

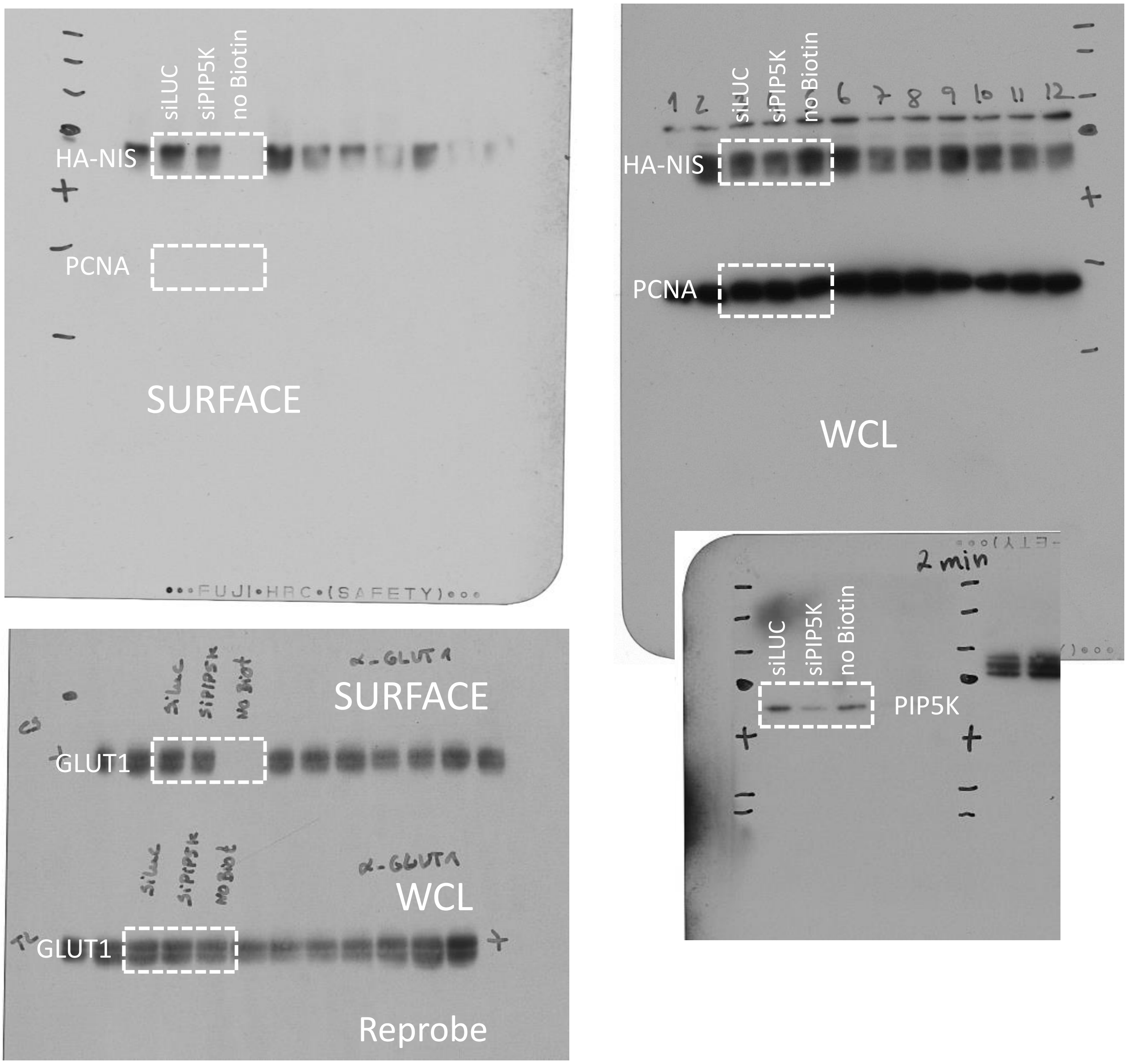

siLUC
siPIP5K
no Biotin
HA-NIS
PCNA
SURFACE
FUJI HRC (SAFETY)
1 2
6 7 8 9 10 11 12
WCL
2 min
PIP5K
GLUT1
Reprobe

Figure 8C

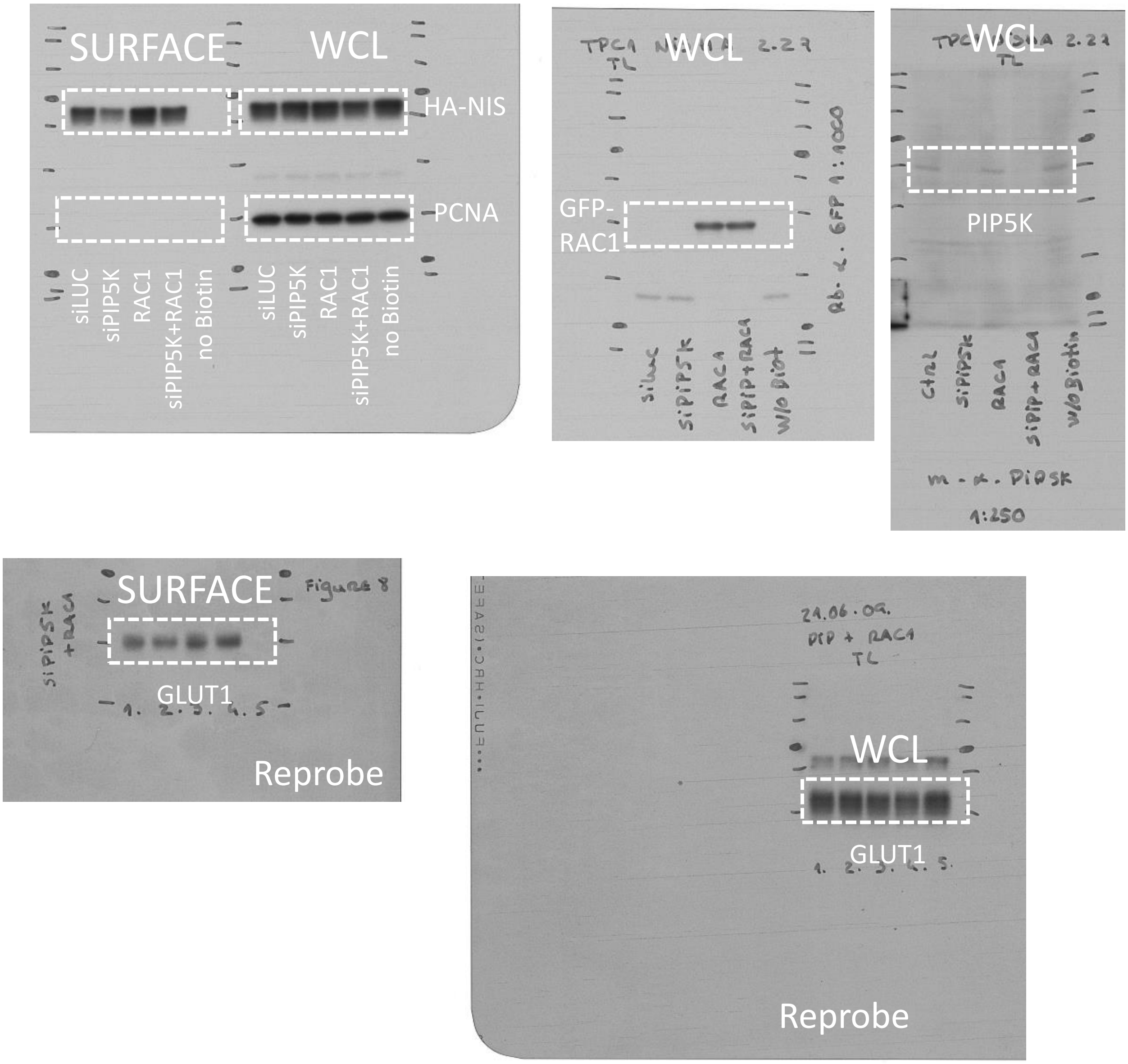
SURFACE
WCL
HA-NIS
PCNA
siLUC
siPIP5K
RAC1
siPIP5K+RAC1
no Biotin
siLUC
siPIP5K
RAC1
siPIP5K+RAC1
no Biotin
WCL
GFP-RAC1
WCL
PIP5K
SURFACE
GLUT1
Reprobe
WCL
GLUT1
Reprobe

Figure 9A

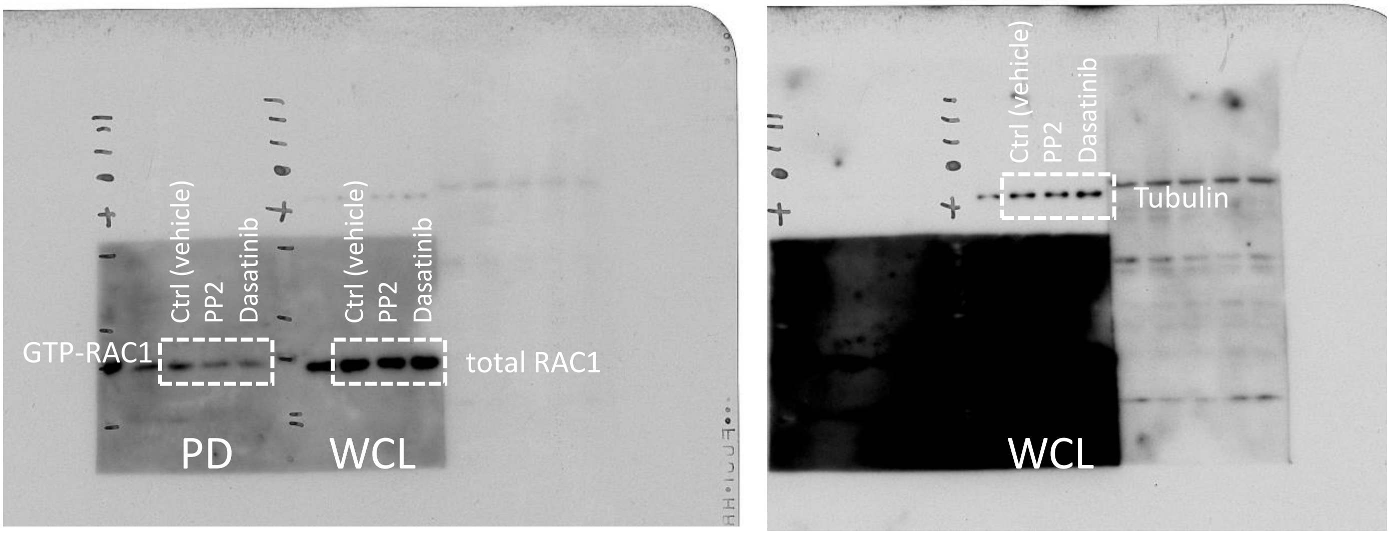

Figure 9B

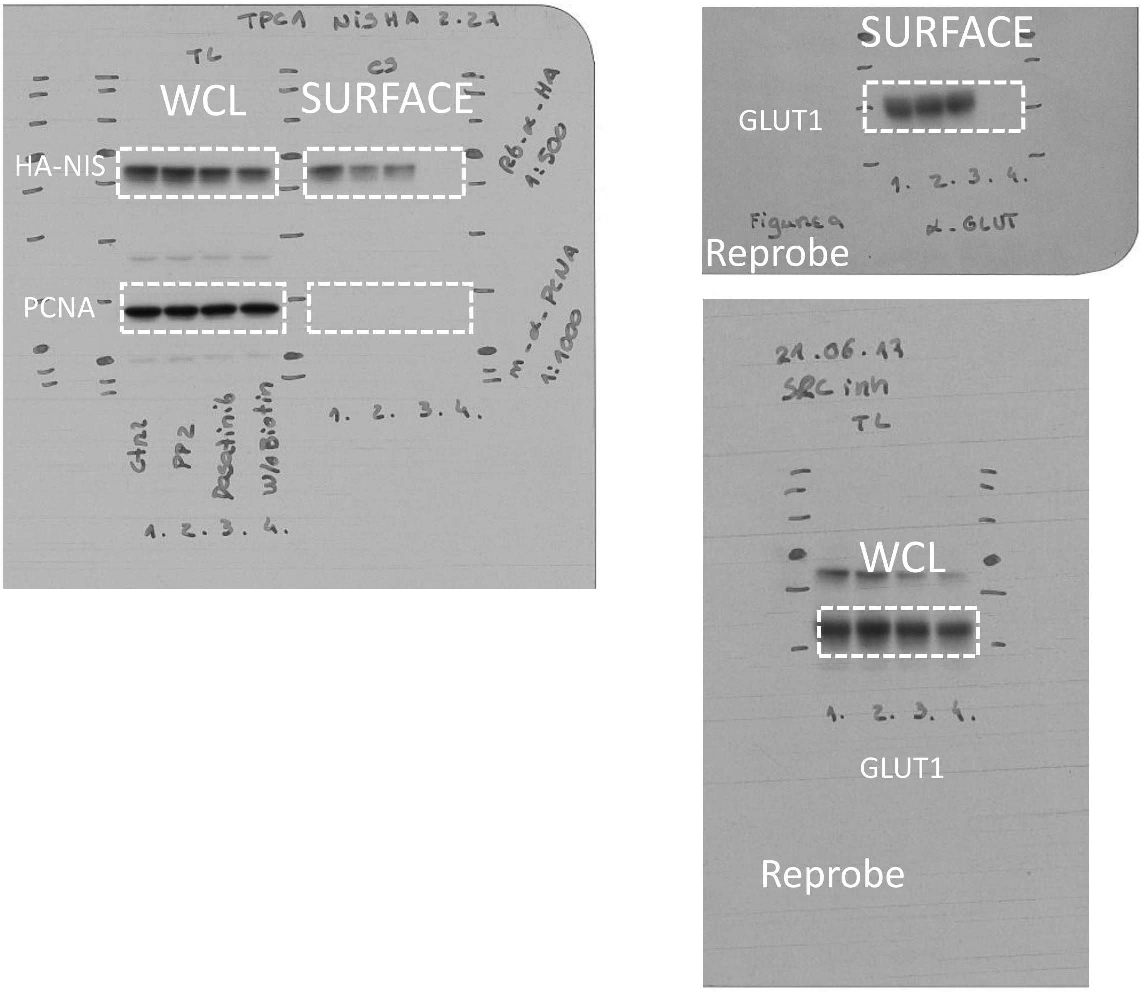

Supplemental Figure S6A

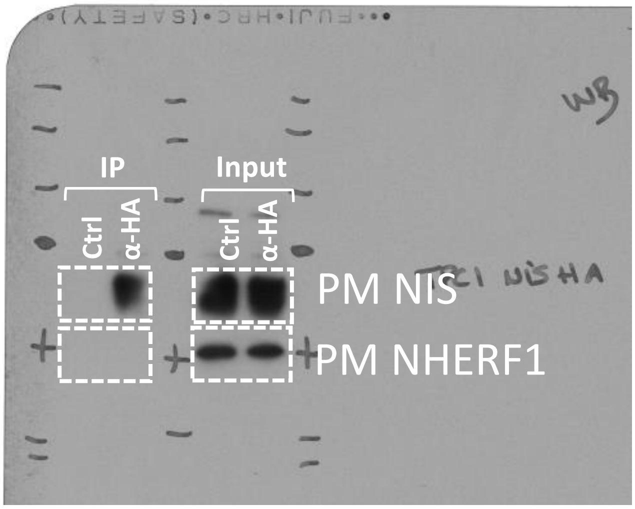

Supplement: Supplementary file 1 [file cancers-13-05460-s001.zip › Faria et al_2021_Original WB films_2nd revision__F.pdf]
