# Supplementary material for: Analysis of NIS Plasma Membrane Interactors Discloses Key Regulation by a SRC/RAC1/PAK1/PIP5K/EZRIN Pathway with Potential Implications for Radioiodine Re-Sensitization Therapy in Thyroid Cancer
Source: Cancers (Basel). 2021 Oct 30;13(21):5460. doi: 10.3390/cancers13215460 (PMC8582450; doi:10.3390/cancers13215460)
Supplement: Supplementary file 1 [file cancers-13-05460-s001.zip › Faria et al_2021_Supplementary_Figures_Final_2nd revision_F.pdf]

Supplementary Figures to

Analysis of NIS plasma membrane interactors discloses key regulation by a SRC/RAC1/PAK1/PIP5K/EZRIN pathway with potential implications for radioiodine re-sensitization therapy in thyroid cancer

Márcia Faria[1,4,5], Rita Domingues[1,2], Maria João Bugalho[1,3], Ana Luísa Silva[1,2,3,6], Paulo Matos[4,5,6,*]

[1] Serviço de Endocrinologia, Diabetes e Metabolismo do CHULN-Hospital Santa Maria, 1649-028 Lisboa, Portugal.
[2] ISAMB-Instituto de Saúde Ambiental, Faculdade de Medicina da Universidade de Lisboa, 1649-028 Lisboa, Portugal.
[3] Faculdade de Medicina da Universidade de Lisboa, 1649-028 Lisboa, Portugal.
[4] BioISI-Biosystems and Integrative Sciences Institute, Faculdade de Ciências da Universidade de Lisboa, 1749-016 Lisboa, Portugal.
[5] Departamento de Genética Humana, Instituto Nacional de Saúde Doutor Ricardo Jorge, 1649-016 Lisboa, Portugal.
[6] Co-senior authors

* Corresponding author

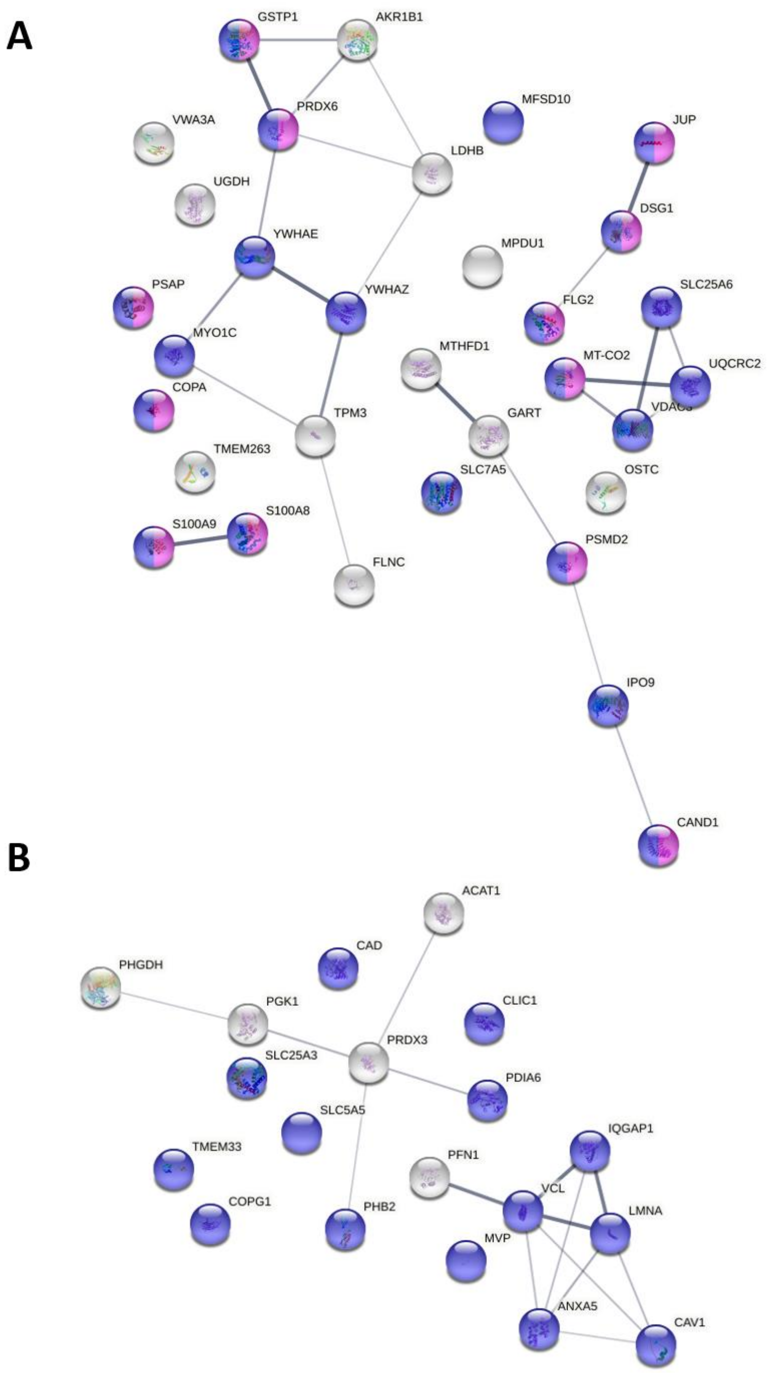

Figure S1. STRING analysis of the WC-NIS dataset. (A) STRING-generated network depicting predicted associations between the 33 high confidence protein hits detected by MS as co-precipitating exclusively with NIS isolated from whole cell lysates (WC-NIS dataset). Network nodes represent the identified proteins. The grey lines connecting two nodes represent protein associations extrapolated from textmining-, experimental- and database-collected evidence. The thickness of the lines is proportional to the degree of confidence for the predicted association between nodes. Blue and pink overlay colors indicate proteins under the most represented "transport" and "secretion" GO terms, respectively (see also Table 1). **(B)** STRING network generated as in (A), depicting predicted associations between the 19 proteins common to the WC-NIS and PM-NIS high confidence protein datasets. Blue overlay color indicates proteins under the highly enriched "transport" GO terms (see also Table 1).

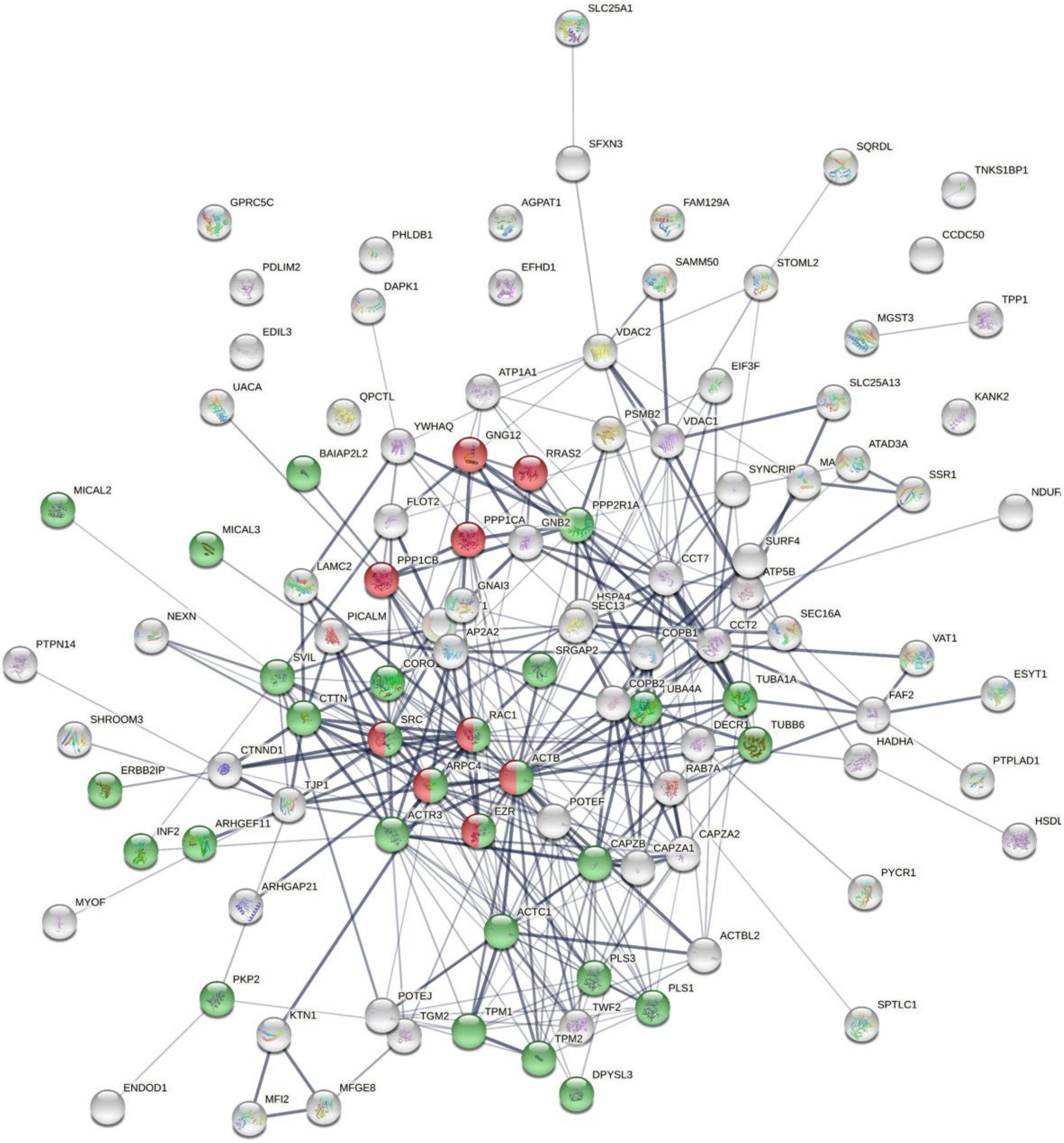

Figure S2. STRING analysis of the PM-NIS dataset. STRING-generated network depicting predicted associations between the 109 high confidence protein hits detected by MS as co-precipitating exclusively with NIS isolated from the plasma membrane (PM-NIS dataset). Network nodes represent the identified proteins. The grey lines connecting two nodes represent protein associations extrapolated from textmining-, experimental- and database-collected evidence. The thickness of the lines is proportional to the degree of confidence for the predicted association between nodes. Green overlay color indicates the 29 proteins under the most represented "cytoskeleton organization" GO term (see also Table 1). Red overlay color highlights the 10 proteins annotated as belonging to the "Regulation of actin cytoskeleton" pathway in the KEGG database.

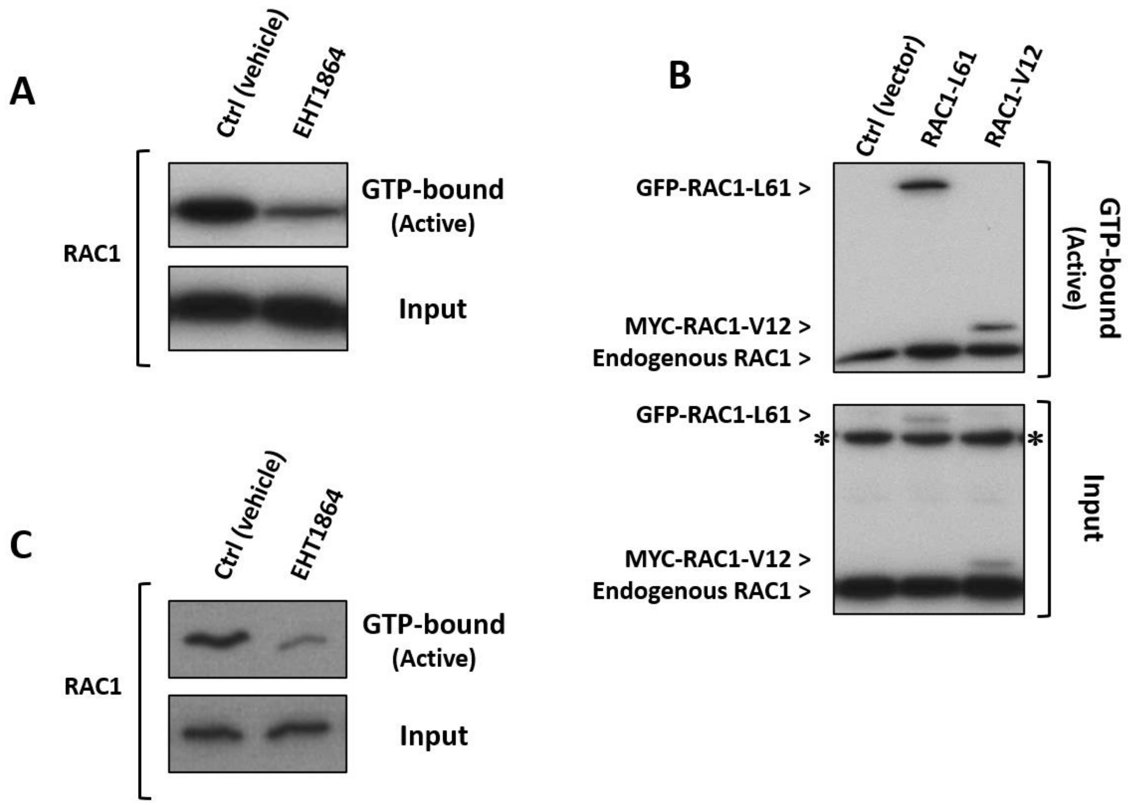

Figure S3. Assessment of RAC1 activation status in HA-NIS-TPC1 and PCCL3 cells. HA-NIS-TPC1 **(A and B)** or PCCL3 **(C)** cells were either treated with vehicle (Ctrl) or 50 µM EHT1864 for 1 hour (A and C) or transiently transfected with empty vector (Ctrl), GFP-RAC1-L61 or MYC-RAC1-V12 expressing constructs (B)**.** RAC1 activation status was then assessed by monitoring the active, GTP-bound fraction of RAC1 proteins by CRIB domain pull-down assay. Total (input) and GTP-bound RAC1 levels were assessed by western blot using an anti-RAC1 primary antibody, which detects both endogenous RAC1 and the transfected RAC1 mutants, as indicated. Note that the "*****" in panel (B) denotes an unspecific band labeled by the anti-RAC1 antibody in whole cell lysates, that does not correspond to RAC1 and, thus, is not captured by the CRIB-peptide, not appearing in the pull-down immunoblot.

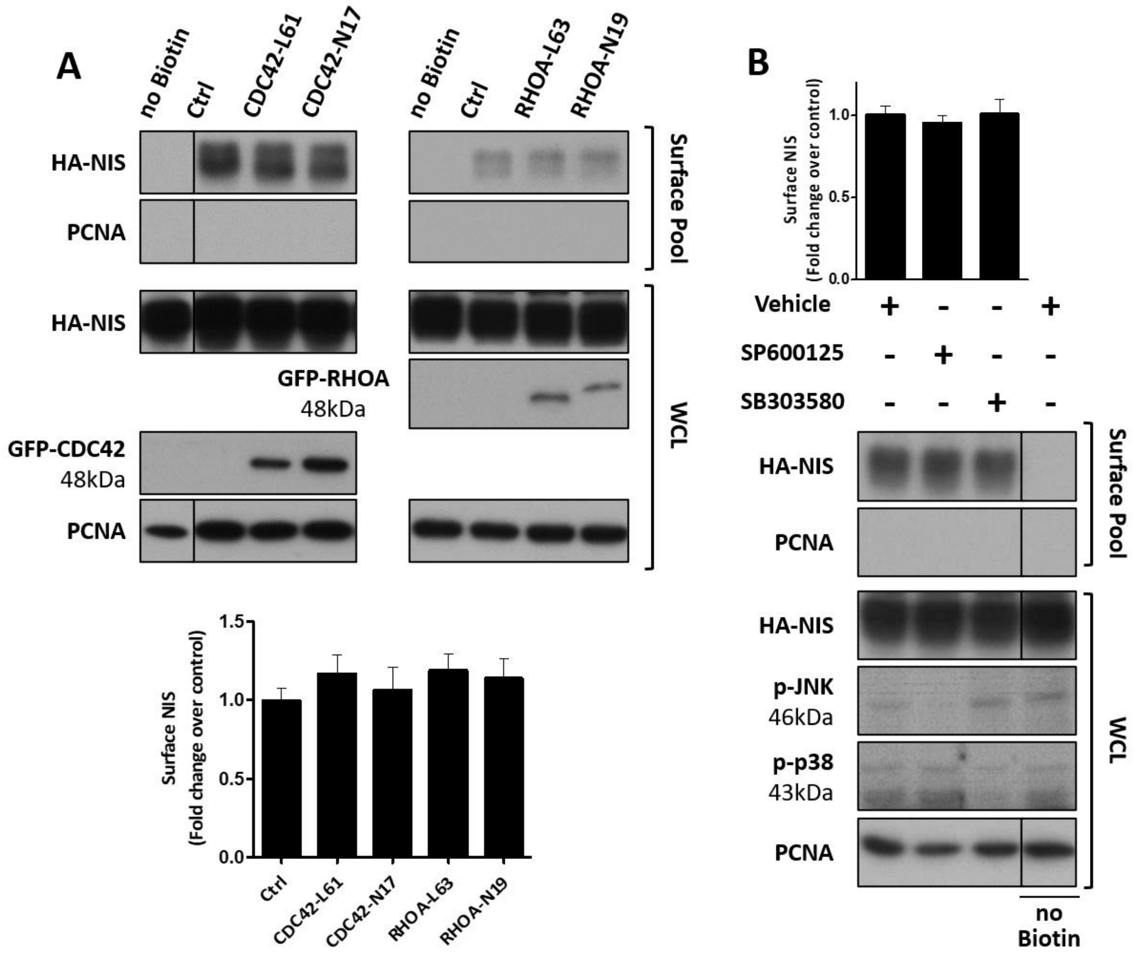

Figure S4. Impact on HA-NIS surface levels of CDC42 and RHOA GTPases, and of RAC1 effector kinases JNK and p38. (A) HA-NIS-TPC1 cells were transiently transfected with empty vector (Ctrl) or either constitutively active (L61/L63) or dominant negative (N17/N19) constructs of CDC42 or RHOA, as indicated, and analyzed by surface protein biotinylation. Surface fractions and the correspondent whole-cell lysates (WCL) analyzed by WB, as indicated. PCNA expression served as loading (WCL) and intracellular protein contamination control (Surface pool). WB bands were quantified by densitometric analysis using ImageJ software. Plotted values are means ± SEM of three independent assays. One-way ANOVA analysis detected no significant differences between treatments ($F = 0.4481$; $P = 0.7718$). **(B)** HA-NIS-TPC1 cells were treated with either vehicle or SP600125 (30 µM for 1 hour) or SB203580 (10 µM for 1 hour) to inhibit JNK and p38 signaling, respectively, and then analyzed by surface protein biotinylation and WB, as in (A). JNK and p38 auto-phosphorylation levels were monitor by WB to confirm inhibition of their activity. Plotted values are means ± SEM of four independent assays. One-way ANOVA analysis detected no significant differences between treatments ($F = 0.2265$; $P = 0.8017$).

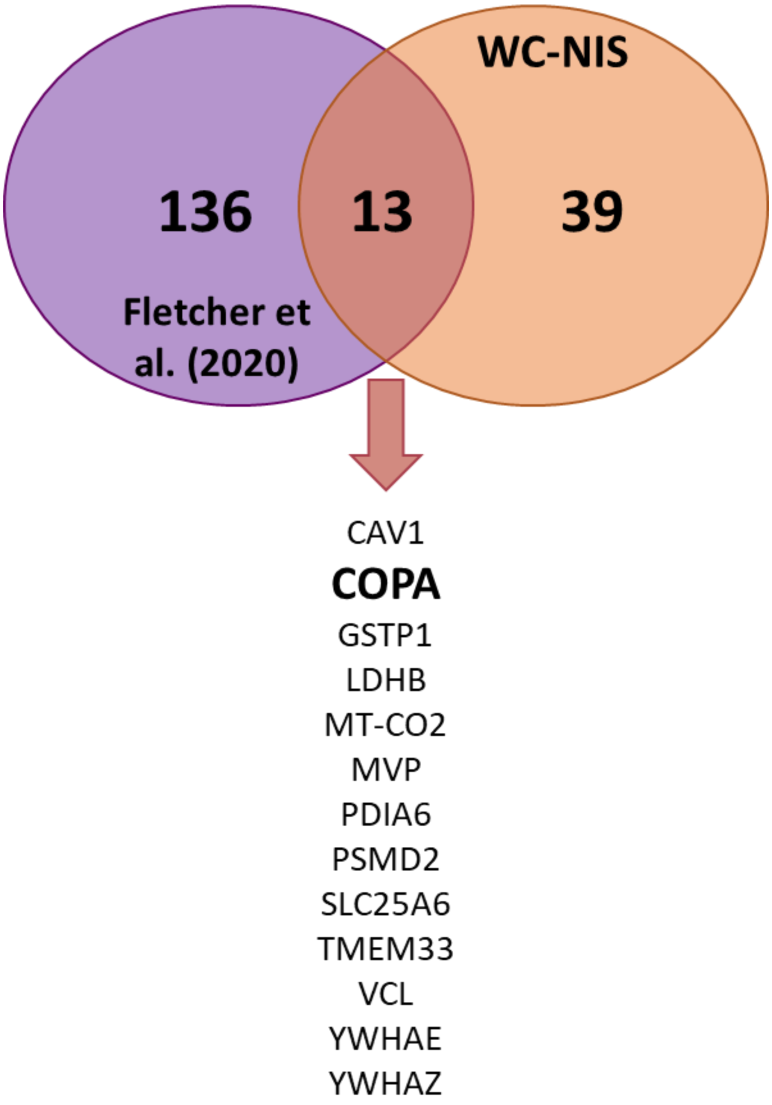

Figure S5. Comparison of the WC-NIS dataset with previously reported putative NIS interactors. Venn diagram depicting the intersection between candidate NIS interactors from our WC-NIS dataset and that published by Fletcher et al. [23]. Listed are the names of the genes coding for the 13 common proteins.

A

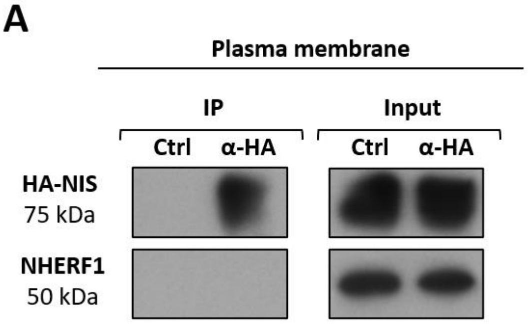

B

Sodium Iodide Symporter a.a. sequence [Homo sapiens]

Accession: AAB17378.1

GI: 1628579

```
Meavetgerptfgawdygvfalmllvstgiglwvglarggqrsaedfftggrrlaalpvglslsasfmsa
vqvlgvpseayryglkflwmclgqllnsvltallfmpvfyrlgltstyeylemrfsravrlcgtlqyiva
tmlytgiviyapalilnqvtgldiwasllstgiictfytavggmkavvwtdvfqvvvmlsgfwvvlargv
mlvggprqvltlaqnhsrinlmdfnpdprsrytfwtfvvggtlvwlsmygvnqaqvqryvacrtekqakl
allinqvglflivssaaccgivmfvfytdcdplllgrisapdqympllvldifedlpgvpglflacaysg
tlstastsinamaavtvedlikprlrslaprklviiskglsliygsacltvaalssllgggvlqgsftvm
gvisgpllgafilgmflpacntpgvlaglgaglalslwvalgatlyppseqtmrvlpssaarcvalsvna
sglldpallpandssrapssgmdasrpaladsfyaisylyygalgtlttvlcgaliscltgptkrstlap
gllwwdlarqtasvapkeevailddnlvkgpeelptgnkkppgflptnedrlfflgqkelegagswtpcv
ghdggrdqqetnl
```

Legend (domain coordinates adapted from DOI:10.1210/jcem.84.8.5871):

Extracellular loop

Transmembrane domain

Intracellular loop

Putative FERM-binding motive

Figure S6. Analysis of potential mechanisms for the NIS-EZRIN interaction. (A) Input lysates and NIS-PM immunoprecipitated protein complexes were analyzed by WB using a specific anti-NHERF1 antibody. Input and IP HA-NIS levels were also detected using a mouse anti-HA primary antibody, to confirm immunoprecipitation efficiency. **(B)** Analysis of human NIS protein sequence revealed two putative FERM-interacting xYxV motifs near the interfaces between NIS 2nd cytoplasmic loop and 4th transmembrane domain, and between NIS 7th transmembrane domain and 4th cytoplasmic loop (green highlight).
